# Fluctuations in *spo0A* Transcription Control Rare Developmental Transitions in *Bacillus subtilis*

**DOI:** 10.1371/journal.pgen.1002048

**Published:** 2011-04-28

**Authors:** Nicolas Mirouze, Peter Prepiak, David Dubnau

**Affiliations:** 1 Public Health Research Center, New Jersey Medical School, Newark, New Jersey, United States of America; 2 Department of Microbiology and Molecular Genetics, New Jersey Medical School, Newark, New Jersey, United States of America; Agency for Science, Technology, and Research, Singapore

## Abstract

Phosphorylated Spo0A is a master regulator of stationary phase development in the model bacterium *Bacillus subtilis*, controlling the formation of spores, biofilms, and cells competent for transformation. We have monitored the rate of transcription of the *spo0A* gene during growth in sporulation medium using promoter fusions to firefly luciferase. This rate increases sharply during transient diauxie-like pauses in growth rate and then declines as growth resumes. In contrast, the rate of transcription of an rRNA gene decreases and increases in parallel with the growth rate, as expected for stable RNA synthesis. The growth pause-dependent bursts of *spo0A* transcription, which reflect the activity of the *spo0A* vegetative promoter, are largely independent of all known regulators of *spo0A* transcription. Evidence is offered in support of a “passive regulation” model in which RNA polymerase stops transcribing *rRNA* genes during growth pauses, thus becoming available for the transcription of *spo0A*. We show that the bursts are followed by the production of phosphorylated Spo0A, and we propose that they represent initial responses to stress that bring the average cell closer to the thresholds for transition to bimodally expressed developmental responses. Measurement of the numbers of cells expressing a competence marker before and after the bursts supports this hypothesis. In the absence of ppGpp, the increase in *spo0A* transcription that accompanies the entrance to stationary phase is delayed and sporulation is markedly diminished. In spite of this, our data contradicts the hypothesis that sporulation is initiated when a ppGpp-induced depression of the GTP pool relieves repression by CodY. We suggest that, while the programmed induction of sporulation that occurs in stationary phase is apparently provoked by increased flux through the phosphorelay, bet-hedging stochastic transitions to at least competence are induced by bursts in transcription.

## Author Summary

A hallmark of the intensively studied model organism *Bacillus subtilis* is its ability to enter developmental pathways: forming spores, acquiring the ability to take up environmental DNA, and the formation of biofilms. These pathways are dependent on the transcription factor Spo0A. All are expressed heterogeneously across populations of cells and exhibit characteristic rates of transition to the developmental pathways depending on environmental signals. We have monitored the rate of transcription of *spo0A* during growth and have detected unexpected fluctuations that correlate with pauses in the growth rate. We present support for a model in which the release of RNA polymerase from transcription of ribosomal RNA genes during the growth pauses permits increased transcription of *spo0A*. We show that these bursts in transcription increase the still-rare probability of transition to the transformable state, suggesting that this transition is limited by the transcription rate of *spo0A*. In contrast, it has been shown that the programmed development of spores is determined by the rate of phosphorylation of Spo0A. Thus there are two modes of developmental transition. We also show that a popular hypothesis for the initiation of spore formation by release of repression by the protein CodY is incorrect.

## Introduction

In response to nutritional deprivation and high population density, the model bacterium *Bacillus subtilis* can embark on several developmental programs leading to sporulation, cannibalism, biofilm formation and genetic competence [1]–[4]. These exquisitely regulated responses anticipate starvation, initiating before crucial metabolic pools are exhausted.

Although the various developmental programs of *B. subtilis* require the participation of specific signal transduction genes, many regulators are shared, presumably detecting common signals and coordinating the responses. Most notable among the shared regulators is Spo0A, which plays an essential role in each of the developmental pathways listed above. The concentration of Spo0A∼P increases gradually in a controlled manner as cells enter stationary phase, binding to target promoters as an activator or repressor of transcription, with a hierarchy governed by DNA-binding affinities [5]. High affinity promoters respond early and with kinetic heterogeneity, i.e. some cells respond earlier than others [6], [7]. This probably reflects kinetic heterogeneity in the accumulation of Spo0A∼P, which plays an important biological role [7]–[9]. The upstream signals and all of the genes and pathways that control the synthesis of Spo0A protein and regulate its phosphorylation and dephosphorylation have not been completely described. In important reports it has recently been shown that the rate limiting process in the early stages of sporulation is the flux of phosphate through the phosphorelay, rather than the synthesis of Spo0A protein [7], [10].

Apart from posttranscriptional regulation involving phosphorylation and dephosphorylation, the transcriptional control of *spo0A* is complex. During growth, *spo0A* is transcribed from the Pv promoter, which is dependent on the major housekeeping sigma factor, SigA. As cultures enter stationary phase in media that support sporulation, transcription from the downstream Ps promoter takes over, dependent on the minor sigma factor, SigH [11]–[13]. The circuitry involved in this promoter switching is incompletely described, although it is known that before stationary phase the transcription of *sigH* is repressed by AbrB [14], which is in turn repressed by Spo0A∼P [15]. This indirect positive feedback circuit is activated as cells enter stationary phase, perhaps by posttranscriptional regulation of SigH [16], [17]. Although the positive feedback may be kinetically limited by the availability of Spo0A∼P, it is likely that an understanding of both transcriptional and posttranscriptional regulation of Spo0A∼P synthesis will prove essential for the complete kinetic description mentioned above.

Competence, sporulation and the early steps in biofilm formation are bimodally expressed in clonal populations [3], [18]. This population heterogeneity has been ascribed to cell-to-cell variability (noise) in the expression of critical regulatory proteins [19], most likely Spo0A∼P in the case of spore formation. In competence, intrinsic noise in the expression of the *comK* gene determines which cells transition to the competent state [20], [21]. Remarkably, the probability of the transition to competence increases as cultures enter stationary phase and decreases thereafter. In other words the average cell is brought closer to and then moved away from a transition threshold [21], [22].

Classical work by E. Freese and colleagues demonstrated that sporulation could be initiated by the experimental manipulation of the GTP pool and suggested that a drop in this pool is normally responsible for triggering sporulation [23], [24]. One pathway leading to a decrease in the GTP pool, particularly in response to amino acid deprivation, involves the so-called “stringent” response [25]. In both *B. subtilis* and *Escherichia coli*, nutrient deprivation elicits the synthesis of the alarmones pppGpp and ppGpp. In *B. subtilis*, ppGpp (used here to represent both alarmones) inhibits IMP dehydrogenase and hence the synthesis of GTP [26]. As a result, the precursor inosine monophosphate is available for AMP synthesis and ATP increases in concentration as the amount of GTP decreases. These considerations led to a model in which starvation causes the accumulation of ppGpp, a decrease in the GTP pool and thus the initiation of sporulation [27], [28]. Although the mechanism by which a decrease in GTP triggers spore development is not known, it has been suggested that decreased GTP results in the relief of *spo0A* repression by CodY, which requires GTP as a co-repressor [29]–[31]. As noted above, strong evidence for an alternative model has been presented; suggesting that the critical initiating event is the flux of phosphate through the phosphorelay possibly caused by the synthesis of the histidine kinase KinA beyond a threshold level [7], [10].

We have examined the transcription of *spo0A* during growth and stationary phase and its relationship to the stringent response. To do this we have utilized firefly luciferase as a real-time reporter for the *rate* of transcription. With this tool, which is new to the *Bacillus* community, we show that the transcription of *spo0A* varies dramatically, exhibiting bursts during the growth phase in commonly used media. The onset of each burst in transcription corresponds to a transient decrease in the growth rate of the culture and each decline in the transcription rate from P*spo0A* corresponds to an increase in growth rate. As expected, transcription from an rRNA promoter exhibits the opposite behavior, slowing and speeding up in concert with the growth rate. Experimental evidence is presented in support of a “passive regulation” mechanism, in which RNA polymerase stops transcribing the ribosomal RNA genes during the growth arrests, thus becoming available for the increased transcription of *spo0A* as well as of certain other genes. We have studied the major fluctuations in *spo0A* promoter activity that occur during the growth phase and show that these are not influenced in a major way by factors known or proposed to act at P*spo0A* and are not eliminated in a strain that no longer synthesizes ppGpp. We propose that the increased *spo0A* transcription that accompanies a decreased growth rate is a stress response that serves to increase the probabilities that cells will enter developmental pathways. We confirm this model for competence. Our data imply that the synthesis of Spo0A∼P is limited by transcription from the vegetative promoter of *spo0A* during exponential growth. In contrast to these transcription rate bursts, the programmed increase in *spo0A* transcription that takes place as a culture approaches and enters stationary phase is delayed by mutational inactivation of ppGpp synthesis. However, we show that a popular model for the initiation of sporulation by CodY derepression caused by ppGpp accumulation is unlikely to be correct.

## Results

### Use of firefly luciferase as a transcriptional reporter in *B. subtilis*

To monitor transcription, we have used promoter fusions to firefly (*Photinus pyralis*) luciferase with cultures growing in the presence of luciferin in 96-well plates. Luciferase catalyzes the oxidation of luciferin in the presence of cellular ATP to emit light, which can be measured in a plate reader equipped for luminometry. The Claverys lab has previously used firefly luciferase to study gene expression in *Streptococcus pneumoniae* [32], [33] but to our knowledge this reporter has not been widely employed in bacteriology. For our experiments, growth was measured in a temperature controlled plate reader by the measurement of optical density at 600 nm. Readings of optical density and light output were taken approximately every 1.5 minutes, providing a sensitive and highly time-resolved real-time determination of both promoter activity and growth. Firefly luciferase is unstable in *B. subtilis*, with a half-life of about 6 minutes (Figure S1C and S1D). Light emission therefore declines when the transcription rate drops and the luminometry readings (given in Relative Luminescence Units (RLUs)) reflect the *rate* of transcription. The changes in light output we have determined during growth are not caused by changes in luciferin permeability or by changes in the ATP pool (Figure S1A, S1B and Text S1). A further important feature of the method is its remarkable reproducibility (Figure S2). The use of firefly luciferase has revealed changes in gene expression that would not be detectable using reporters that accumulate with time.

### Transcription of *spo0A* and *spoIIG*

**Figure 1:**
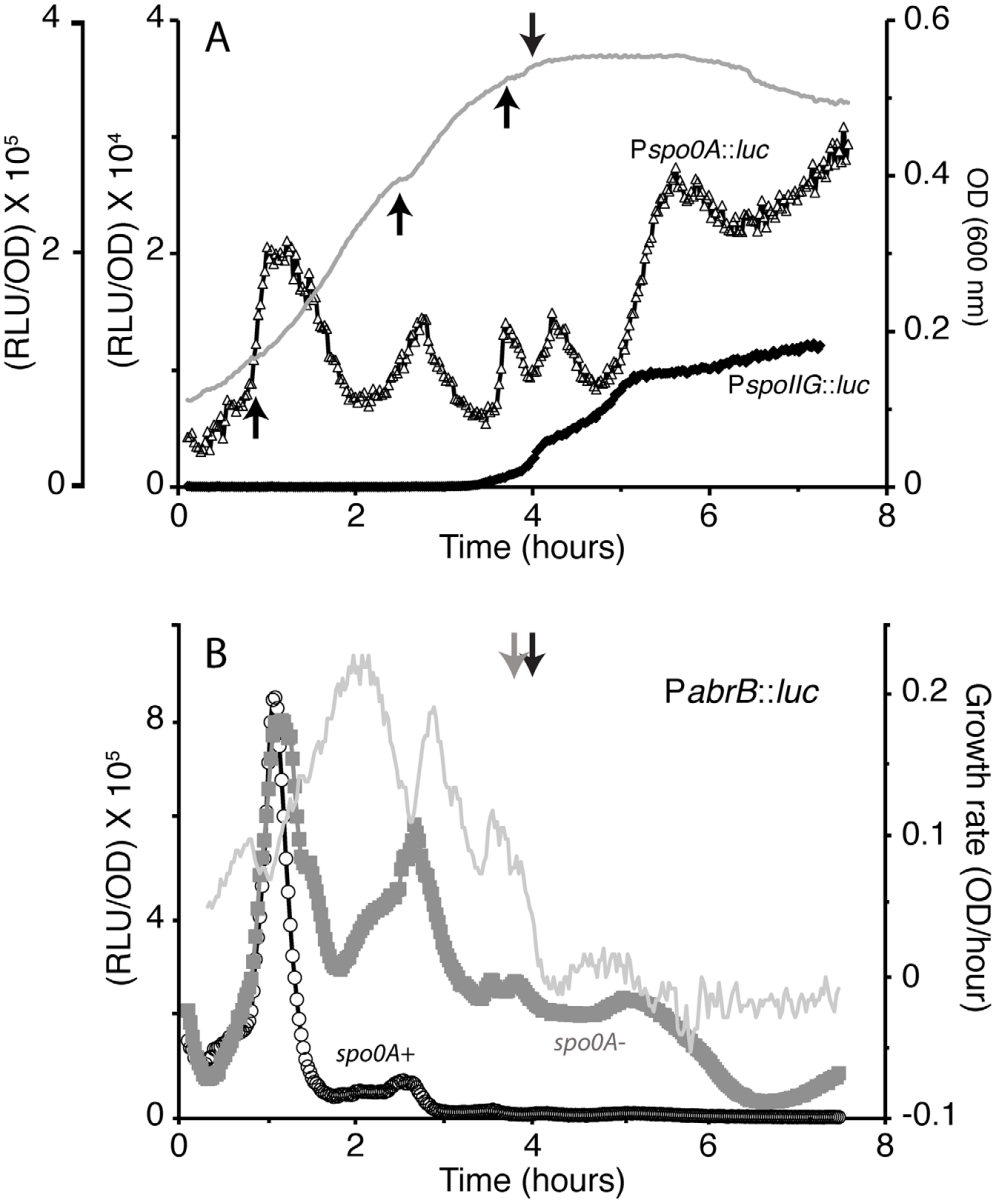
Growth and transcription from *spoIIG*, *spo0A*, and *abrB* promoter fusions to *luc* in DSM medium. (A) The gray curve depicts growth measured by optical density at 600 nm. The gray line connects the OD points, which are not visible, but were taken every 1.5 minutes. The arrows point to inflection points in the growth curve. The open triangles show relative luminescence readings corrected for OD for the *spo0A* promoter and the heavy line shows the same for the *spoIIG* promoter. The ordinate scale to the left (×10^5^) refers to the *spoIIG* promoter. The growth curves of these two strains are nearly identical (not shown). T_0_ is indicated by the downward pointing arrow. (B) Growth rate and transcription from the *abrB* promoter. Strains expressing *luc* from a promoter fusion to *abrB* were grown in DSM. The open circles indicate results for the *spo0A* ^+^ background and the solid gray squares for a *spo0A* strain. The growth rate was numerically determined and is indicated by the gray line. Slightly negative values for growth rate indicate some lysis that occurred after T_0_. T_0_ is indicated by a downward pointing arrow for each strain, black for the *wild type* and grey for the *spo0A* mutant.

Figure 1A shows the results of an experiment in which strains carrying fusions of either the *spo0A* and *spoIIG* promoters to the luciferase (*luc*) coding sequence are grown in a standard medium supporting sporulation (DSM) [34]. Both fusions were constructed by single crossover integration into the corresponding chromosomal loci, ensuring that the *luc* reporter has been placed under control of all the relevant upstream regulatory sequences and that the wild-type *spo0A* locus is undisturbed. The transcription rate from the *spoIIG-luc* promoter increased just before the transition from exponential growth to the stationary phase (T_0_), followed by further step-wise increases after T_0_. Restriction of *spoIIG* transcription to these late growth stages is expected for this sporulation-specific gene that is only transcribed in the presence of a relatively high concentration of Spo0A∼P [35]. In contrast, the transcription rate of *luc* fused to P*spo0A* exhibited 5 prominent bursts, two of which occurred during the growth phase, followed by a third at T_0_, a fourth about 20 minutes later and a fifth more prominent increase in transcription rate beginning at about T_1_ and followed by a sustained rise after T_2_. These stationary phase increases in *spo0A* transcription presumably reflect the early stages of global commitment to spore formation. It is noteworthy that the peak transcription rates achieved before T_0_ are appreciable, about half to three-fourths the maximum rate achieved by the fifth burst. Thus substantial but transient transcription of *spo0A* occurs during the growth phase, although the transcription rate from P*spoIIG* is higher than that achieved at any point by P*spo0A* (note the ten-fold difference in scales for the two promoters in Figure 1A). It is worth pointing out that the use of a conventional reporter such as *lacZ* would obscure these dramatic fluctuations in transcription rates because of the stability of ß-galactosidase and because of the inadequate time resolution achievable using *lacZ*.

### Correlation of growth rates and *spo0A* transcription

**Figure 2:**
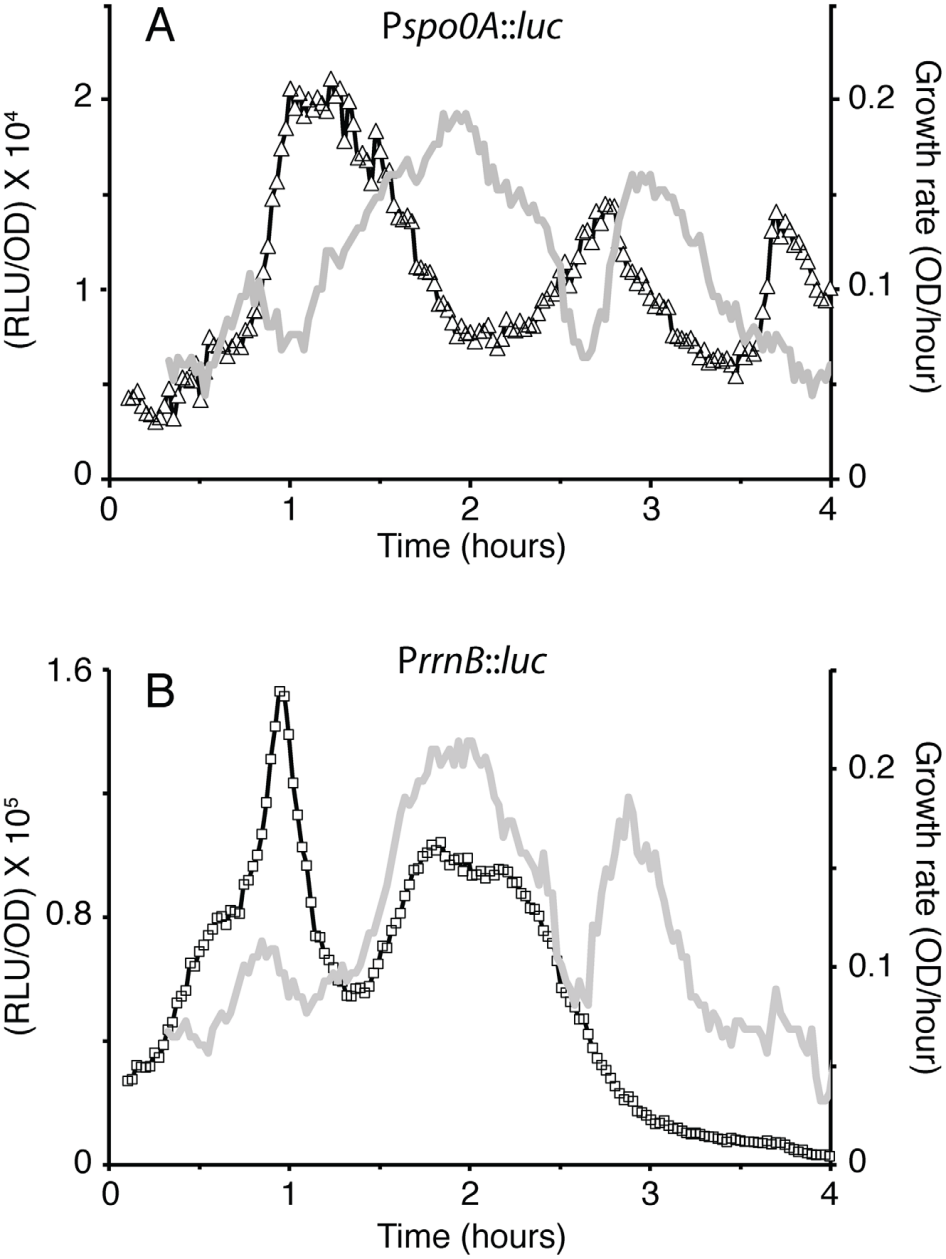
Comparison of *spo0A* and *rrnB* transcription in DSM. (A) The luminescence data from Figure 1 for *spo0A* from the start of the experiment until T_0_ are replotted (open triangles) together with numerically determined values for the growth rate (gray line). (B) Luminescence data from a luciferase fusion to the promoter of *rrnB* is plotted for the first four hours of growth, together with the numerically determined values for the growth rate (gray line).

Each of the first three increases in *spo0A* transcription rate corresponds to a reproducible decrease in the growth rate of the culture, determined from optical density and indicated by the arrows in Figure 1A. Figure 2A presents the OD_600_ data from Figure 1A for the period up to T_0_, in which the numerically determined slope of the growth curve is plotted together with the P*spo0A* transcription rate. It is evident from these data that for the first two inflections that occur during the growth phase, each decrease in growth rate is accompanied by an increase in transcription while each increase in the rate of growth is accompanied by a decrease in the rate of transcription; the phases of the two rate curves are displaced by approximately 180 degrees.

This experiment was repeated in competence medium [36] and in Luria-Bertani broth, which do not support sporulation (not shown). As expected, the expression of *spoIIG* was undetectable in these media. Although the patterns of growth and *spo0A* transcription in the three media were distinct, in each case characteristic growth rate inflections were observed and decreasing growth rate was accompanied by a transient increase in the rate of *spo0A* transcription. In these media sustained increases during stationary phase and at T_0_ were not observed. We interpret these data as indicating that in these three media, growth undergoes a number of characteristic and reproducible pauses related to growth-induced changes in the composition of the medium and to adaptation to these changes, a phenomenon resembling diauxie. Although we may imagine that the growth medium changes independently influence both the growth rate and the *spo0A* promoter it seems much more likely that these changes influence the growth rate, which then influences the *spo0A* promoter. This conclusion is based on the remarkable correspondence of growth rate pauses and transcriptional bursts that occurred consistently throughout these studies. Whatever the nature of the medium changes, they do not result in obvious cell-cycle synchrony, as demonstrated by microscopic observation of fixed cells taken during a growth pause (not shown).

### *rrnB* and *spo0A* respond differently to the growth rate pauses

We wondered whether the observed fluctuations in *spo0A* transcription were promoter-specific or whether they reflected global changes in the initiation of transcription or even changes in the rates of mRNA elongation or in the translation of luciferase. Transcription from ribosomal RNA promoters characteristically decreases together with growth rate upon starvation or down-shifts in nutritional sources [37]. If the regulation of transcription during the pauses in growth rate were promoter specific, we would expect rRNA transcription to decrease in parallel with the growth rate pauses, exhibiting a behavior opposite to that of *spo0A*. To test this we constructed a fusion of luciferase to the P1 promoter of the *rrnB* promoter (Figure 2B). Comparison of Figure 2A and 2B shows that whereas the transcription rate for P*spo0A* is out of phase with the growth rate, that of P*rrnB* parallels the changes in growth rate, as anticipated. These differing patterns must therefore reflect promoter activity rather than mRNA elongation. For the same reason, we also conclude that the fluctuations we observe reflect transcription and not translation. It is noteworthy that the third increase in growth rate is not accompanied by an increase of *rrnB* transcription. Evidently, as the culture approaches stationary phase in DSM, a regulatory mechanism shuts down *rrnB* transcription.

### Response of *spo0A* transcription to carbon source exhaustion

**Figure 3:**
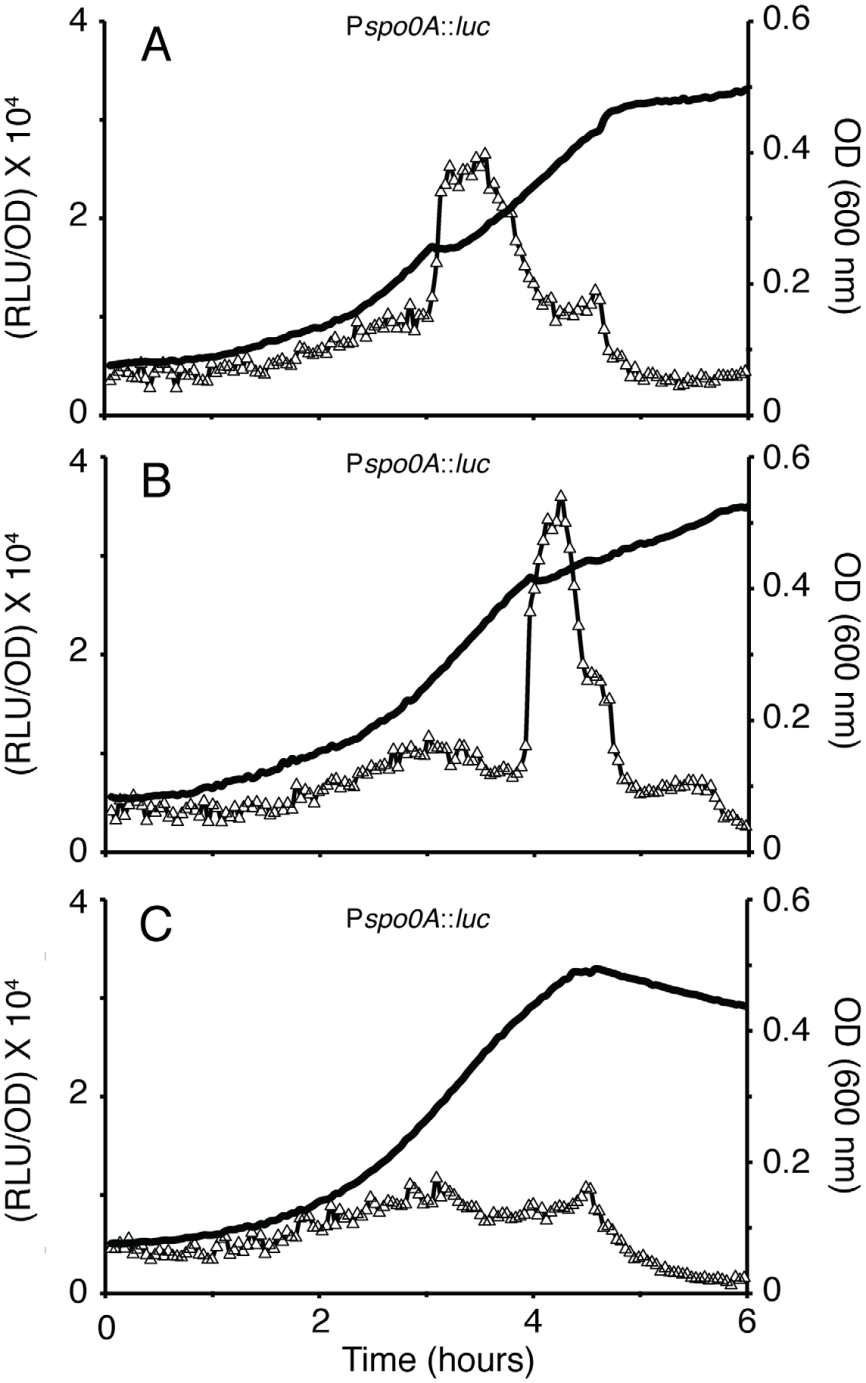
Diauxie and expression from the *spo0A* promoter. A strain expressing *luc* from the *spo0A* promoter (PP530) was grown in S7 minimal medium supplemented with (A) 0.5 mg/ml glucose+2.0 mg/ml arabinose, (B) 1 mg/ml glucose+1.5 mg/ml arabinose and (C) 2.5 mg/ml glucose with no arabinose. The open triangles show P*spo0A-luc* expression and the thick line shows growth.

To further examine the hypothesis that the transcription rate of *spo0A* was responding to diauxie-like pauses in growth, we repeated the classic experiment of J. Monod with *B. subtilis*, utilizing mixtures of glucose and arabinose as the sole carbon sources for cells growing in minimal medium [38]. Figure 3A and 3B show that as expected, pauses in growth occurred when the preferred sugar was exhausted and the positions of the pauses occurred later in growth as the concentration of glucose was increased. Panels A and B also show that the rate of transcription from P*spo0A* increased whenever growth paused due to exhaustion of glucose, indicating that carbon source deprivation induced both growth rate inflections and an increase in the rate of transcription of *spo0A*, although this inverse relationship did not hold when growth ceased during stationary phase. When an excess of glucose was provided (Figure 3C) the growth rate pause was eliminated, as was the corresponding sharp increase in *spo0A* transcription. However, this experiment does not prove that carbon-source exhaustion is responsible for the results in DSM (Figure 1). In fact, when 1% glucose was added to DSM, the shape of the growth curve changed, but three pauses were still evident during growth. In addition, in separate experiments, we have supplemented DSM with each of the 20 amino acids. Again, the shape of the growth curves changed but the growth pauses were still present (not shown). The remainder of this study will report only experiments carried out using DSM, because in this medium, both the growth phase and the stationary phase changes in transcription, potentially related to sporulation could be studied. In what follows we will refer to events occurring prior to T_0_ as taking place during the growth phase, despite the growth rate pauses.

### Protein dependencies of *spo0A* transcription rate changes

**Figure 4:**
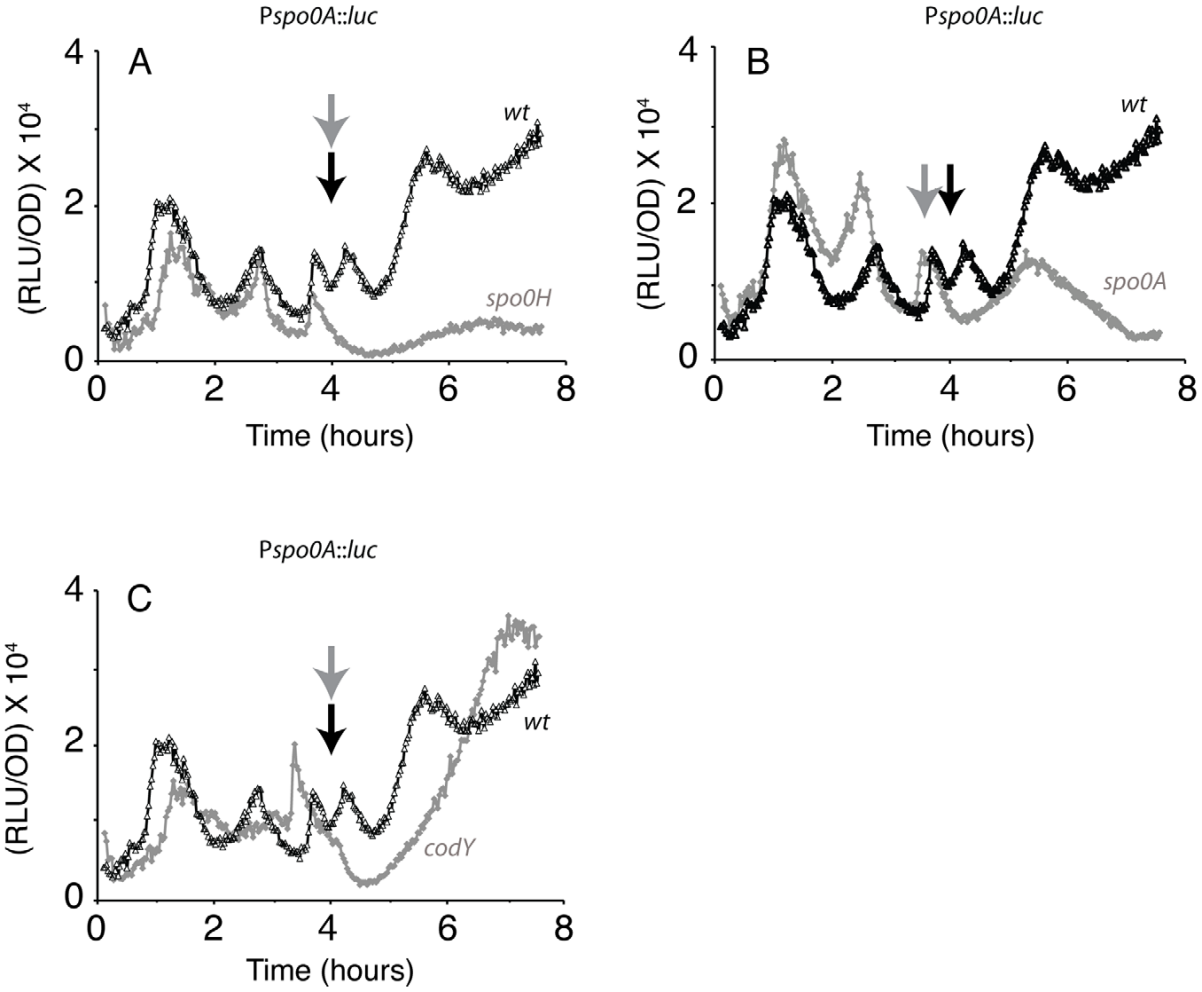
Effects of regulatory gene mutations on *spo0A* transcription. In panels A–C the heavy lines with open triangles show luminescence during growth in DSM from the P*spo0A* promoter. The gray lines show results for knockout mutants in *sigH* (A), *spo0A* (B) and *codY* (C). The black and gray arrows indicate T_0_ for the wild type and mutant strains respectively. In the case of the *spo0A* mutant, T_0_ is reached slightly earlier in accordance with the slightly earlier occurrence of the major bursts.

We next studied the pattern of *spo0A* transcription during growth, to determine the effects of inactivating genes known or at least reported to affect this transcription. As noted above, *spo0A* may be transcribed from either its vegetative (Pv) or sporulation (Ps) promoters [12], [13] and switching from Pv to Ps occurs late in growth [11]–[13]. Figure 4A shows that the inactivation of *sigH* markedly decreased the stationary phase expression of *spo0A* but had only minor effects on the growth stage transcriptional bursts. These results confirm a role for the SigH-dependent Ps promoter after the end of growth, when it is required for sporulation [13]. Importantly, it is clear that the earlier bursts in the rate of *spo0A* transcription reflect changes in transcription from Pv.

Because Spo0A∼P itself acts both positively and negatively on P*spo0A* [39], we next asked whether this molecule was required to establish the pattern of *spo0A* transcription during growth and stationary phase. Only minor differences in the growth curves of a *spo0A* mutant and the wild-type strain were observed, the pauses in growth were not affected by inactivation of *spo0A* (not shown) and the growth phase bursts were clearly not eliminated by disruption of *spo0A* (Figure 4B). The minor changes in the amplitude reflected the auto-activating and –repressing activities of Spo0A. In contrast, expression after T_0_ was markedly decreased. This was expected because the positive effect of Spo0A on its own transcription occurs at the end of the growth phase and is due in part, to activation of SigH [39], [40]. Because the earlier bursts were not dependent on Spo0A, whereas the later ones are, we would expect that inactivation of the kinases that donate phosphoryl groups to the phosphorelay would have minor effects on the early bursts. This was confirmed (Figure S4). Together, these data suggest that although the amplitudes and timing of the bursts are affected in minor ways by the absence of Spo0A∼P during growth, these changes in the *spo0A* transcription rate are not fundamentally dependent on Spo0A, nor on the pathways that deliver phosphoryl groups to this protein.

Other proteins have been reported to affect *spo0A* transcription, either directly or indirectly, including Soj, Spo0J and SinR [41], [42]. Although the validity of these effects or the mode of action of these proteins are in some cases controversial, we have determined the effects of their inactivation on *spo0A* transcription. In fact, inactivation of these factors had minor effects on the relative amplitudes of the *spo0A* transcriptional bursts, but did not disturb the basic pattern or timing of these bursts (Figure S4F–S4I). Because SinR represses the *eps* operon, we studied the effects of *sinR* inactivation in strains also inactivated for *eps* to avoid the severe cell clumping associated with *eps* over-expression [43]. The *sinR* mutation markedly increased the third and fourth bursts, consistent with the reported negative effect of SinR (Figure S4H, S4I). Somewhat surprisingly, we observed that the knockout of *eps* itself slightly increased the second burst and depressed the fifth burst as well as the sustained expression that normally occurs thereafter. Perhaps one or more of the Eps proteins has an unsuspected regulatory role. We conclude that these regulatory proteins are not required for the major fluctuations of *spo0A* transcription during growth.

The results presented so far suggest that in contrast to their effects on the stationary phase transcription of *spo0A*, the major bursts appearing during the growth phase were not eliminated by inactivation of *spo0A*, *sigH*, *sinR*, *spo0J*/*soj*, *kinA*, *kinB*, *kinC*, *kinD* or *kinE*.

### The early bursts of *spo0A* transcription yield phosphorylated Spo0A

Because a relatively high rate of *spo0A* transcription is achieved during the early growth phase, we wondered whether enough phosphorylated Spo0A is produced during these transcriptional bursts to affect the transcriptional program of the cell. The increase in *spo0A* transcription that takes place just before T_0_ clearly results in the synthesis of phosphorylated Spo0A (Spo0A∼P) as demonstrated by the activation of *spoIIG* transcription (Figure 1A). To further explore this question, we utilized a fusion of *luc* to the promoter of *abrB*. This promoter is repressed by the direct binding of Spo0A∼P and should respond quickly, due to its high affinity for Spo0A∼P [15], [44]. Figure 1B shows that transcription from P*abrB* in the *spo0A* ^+^ strain exhibited a major burst in transcription that corresponded to the first growth rate inflection, as well as a much smaller second burst. Notably, the downward segment of the first *abrB* burst occurred just at the point when the first peak of *spo0A* transcription reaches its maximum (Figure 1A). When *spo0A* was inactivated, the rate of transcription from P*abrB* exhibited the same early increase as did the *spo0A^+^* strain, but the following decrease was less profound (Figure 1B). Remarkably, the increased subsequent expression from P*abrB* evident in the *spo0A* strain reveals a series of bursts occurring prior to T_0_, similar to those exhibited by *spo0A* expression, again corresponding to decreases in the growth rate (compare Figure 1A, 1B and Figure 2A). The increase in *abrB* expression when *spo0A* was inactivated shows that the two growth phase spikes in transcription from P*spo0A* produced a burst of Spo0A synthesis and phosphorylation, achieving a sufficiently high concentration of Spo0A∼P to repress P*abrB*. As further evidence that these bursts in *spo0A* transcription are productive we carried out Western blotting for Spo0A protein (see Figure S3). Clearly, the bursts in *spo0A* transcription are followed by increases in the amounts of Spo0A protein in cells growing in both DSM and S7 media. We will comment in the Discussion about a possible cause of the *abrB* bursts in the *spo0A* background.

### The biological role of the transcriptional bursts; transitions to competence during growth

**Figure 5:**
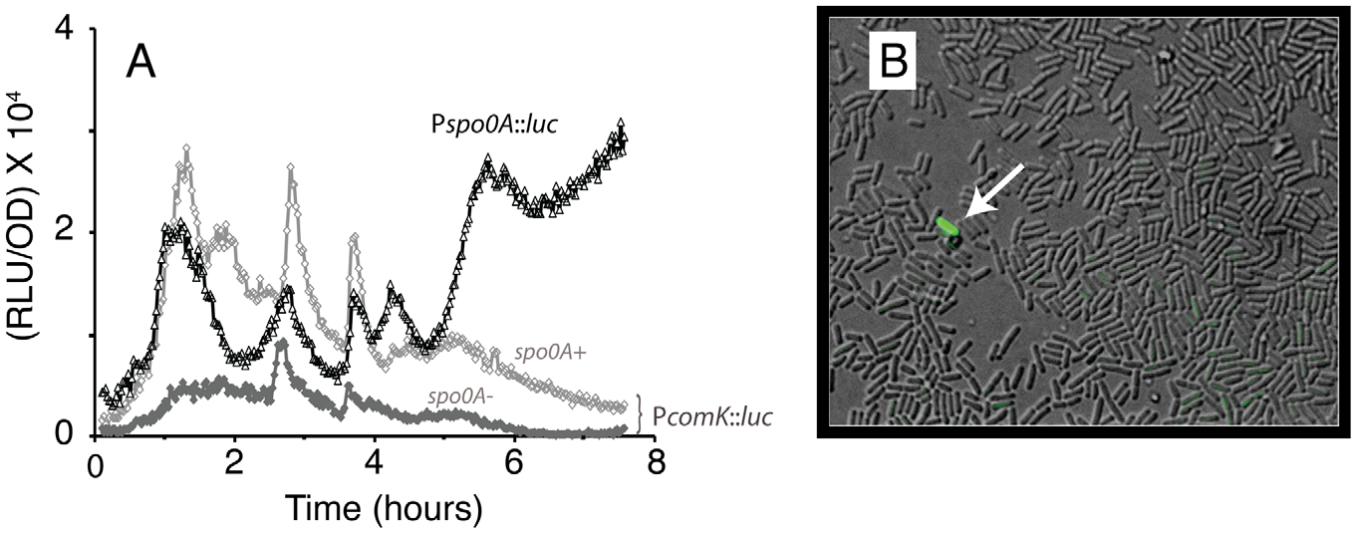
Bursts in *spo0A* transcription result in *comK* bursts. (A) The heavy black line shows expression of *spo0A-luc* during growth in DSM. The light gray line shows the expression of *comK-luc* in DSM and the medium gray line shows the expression of *comK-luc* in the *spo0A* mutant background. (B) A selected microscope field visualized by merging a DIC and a fluorescent image, revealing a single *comK-gfp*-expressing cell among several hundred. The sample was taken from the strain BD5486 (P*spo0A*::*luc* cm, P*comK*-*gfp*::*tet*) growing in the plate reader immediately after the second burst in *spo0A* transcription.

Spo0A∼P is required for competence [36] at a low concentration and at a higher concentration for sporulation [45]. During growth both of these developmental pathways are initiated in rare cells, in contrast to the massive induction that takes place as cultures enter stationary phase in appropriate media. We postulated that the bursts in *spo0A* transcription during the growth phase serve to increase the likelihood that cells will initiate development. As a first test of this possibility, we have determined the transcription pattern of a *PcomK-*luc fusion during growth in DSM in wild type and *spo0A* mutant backgrounds. It has been shown that for cells to enter the competent state, the expression of *comK* must increase so that the average cell is brought closer to the threshold for transition [21]. Figure 5A shows that each burst in *spo0A* transcription is accompanied by a burst in the expression of *comK* and that the expression of this fusion is greatly reduced in the *spo0A* mutant. This suggests that a role for the bursts in *spo0A* transcription is to increase the probability of transition to the competent state. We have tested this hypothesis by microscopically counting *comK-gfp* expressing cells before and after the two major growth phase bursts, in wild type and *spo0A* null mutant strains (Figure 5B). At 0.65 hours from the beginning of growth, prior to the first burst, very few cells (<10^−2^%, 1 out of 15,000) expressed *comK-gfp*. At 3.4 hours, during the declining portion of the second burst, 0.13% (30 out of 23,000) of the cells expressed this marker of development. In the *spo0A* null mutant, no fluorescing cells were detected out of 30,000 and 33,000 cells examined at the early and late times respectively. We conclude that rare cells in a growing population undergo transitions to competence dependent on *spo0A* and that the probability of transition is greater after the two major bursts in *spo0A* transcription. We do not know if the transition probabilities decreased following the bursts, but they clearly responded to the increased concentrations of Spo0A∼P that were produced by the spikes in *spo0A* transcription.

### What causes the growth phase bursts in transcription?

**Figure 6:**
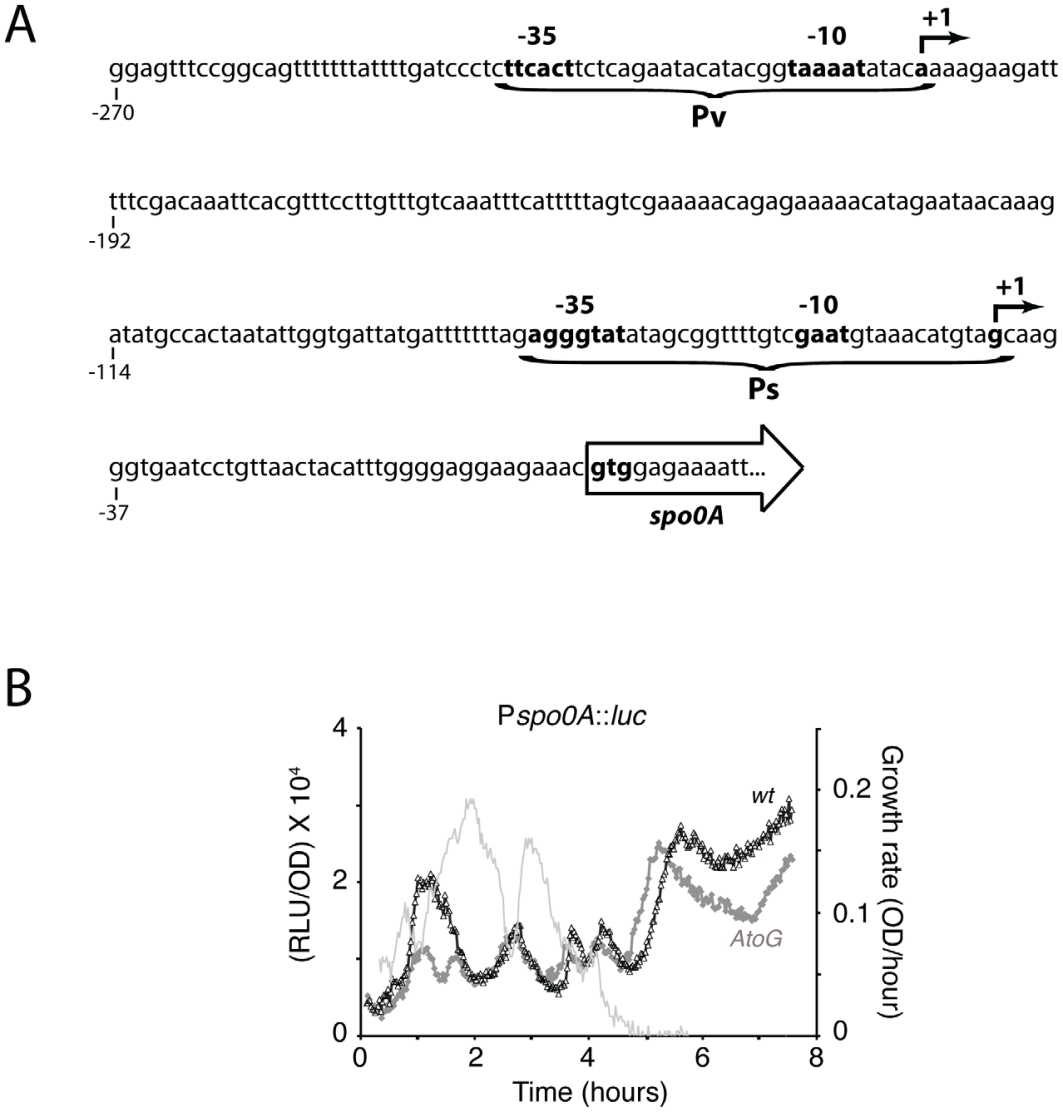
Effects of the AA to GG mutation. (A) Sequence of the promoter region of *spo0A* showing the location of the initiating residues. (B) The dark gray line shows transcription from the Pv promoter of *spo0A* when the AA initiating residues have been changed to GG. The black line shows the same for the wild-type strain and the light gray line shows growth rates for the mutant.

To explain these bursts we considered two hypotheses that are not mutually exclusive. During rapid growth, a major fraction of RNA polymerase (RNAP) molecules is occupied with the transcription of rRNA [46] and as growth slows, less RNAP is so occupied and more becomes rapidly available to other promoters until the cells resume growth. Hence promoters that are limited by the availability of RNAP would almost inevitably exhibit an increase in activity as growth slows. This “passive regulation” model posits that Pv is such a promoter, and hence responds to the release of RNAP during the first growth pause. The second explanation proposes that the growth pause is accompanied by changes in the nucleotide pools that trigger the major burst, because of the identity of the initiating nucleotide for transcription. This hypothesis was suggested by recent work with *B. subtilis*, showing that the identity of the initiating nucleotide for transcription is a factor in the regulation of several genes [37], [47], [48]. In particular, rRNA synthesis in *B subtilis* is decreased by the direct inhibition of GTP synthesis due to the addition of decoynine and by starvation for amino acids, which induces ppGpp synthesis and thus depresses the GTP pool [37]. Altering the start site for *rrnB* transcription from G to A was shown to relieve this inhibition and similar results have been obtained with other promoters that exhibit a stringent response [47], [48]. To explore this idea for *spo0A*, it was first necessary to map the +1 nucleotide for transcription from Pv, which we did using 5′ RACE. The Pv transcriptional start site is at the first A in the sequence AAAAGAAGATTT (Figure 6A). A. Chastanet and R. Losick have obtained the same result using primer extension (personal communication). At the same time we mapped the start site of the Ps promoter, confirming a previous result [49].

To test the roles of the initiating nucleotides, we altered the above sequence to GGAAGAA and incorporated these changes into the Pv promoter of a *spo0A-luc* fusion integrated as before at the *spo0A* locus. The initiating nucleotide hypothesis predicts that in the mutant strain, the bursts in *spo0A* transcription will be absent or at least blunted. Because the initiating nucleotide and RNAP-availability hypotheses are not mutually exclusive, we might still expect to detect a component of regulation that retains the behavior of the wild type Pv promoter. Figure 6B shows that the amplitude of the first burst is indeed reduced in the AA to GG mutant, while the subsequent fluctuations are perhaps slightly blunted. The absence of change in the second burst, which reflects activity at Pv, shows that this promoter is not crippled by the changes we have introduced. Because the bursts still occur, they are not dependent on the identity of the initiating nucleotide or on changes in the nucleotide pools. We will return to this point below.

**Figure 7:**
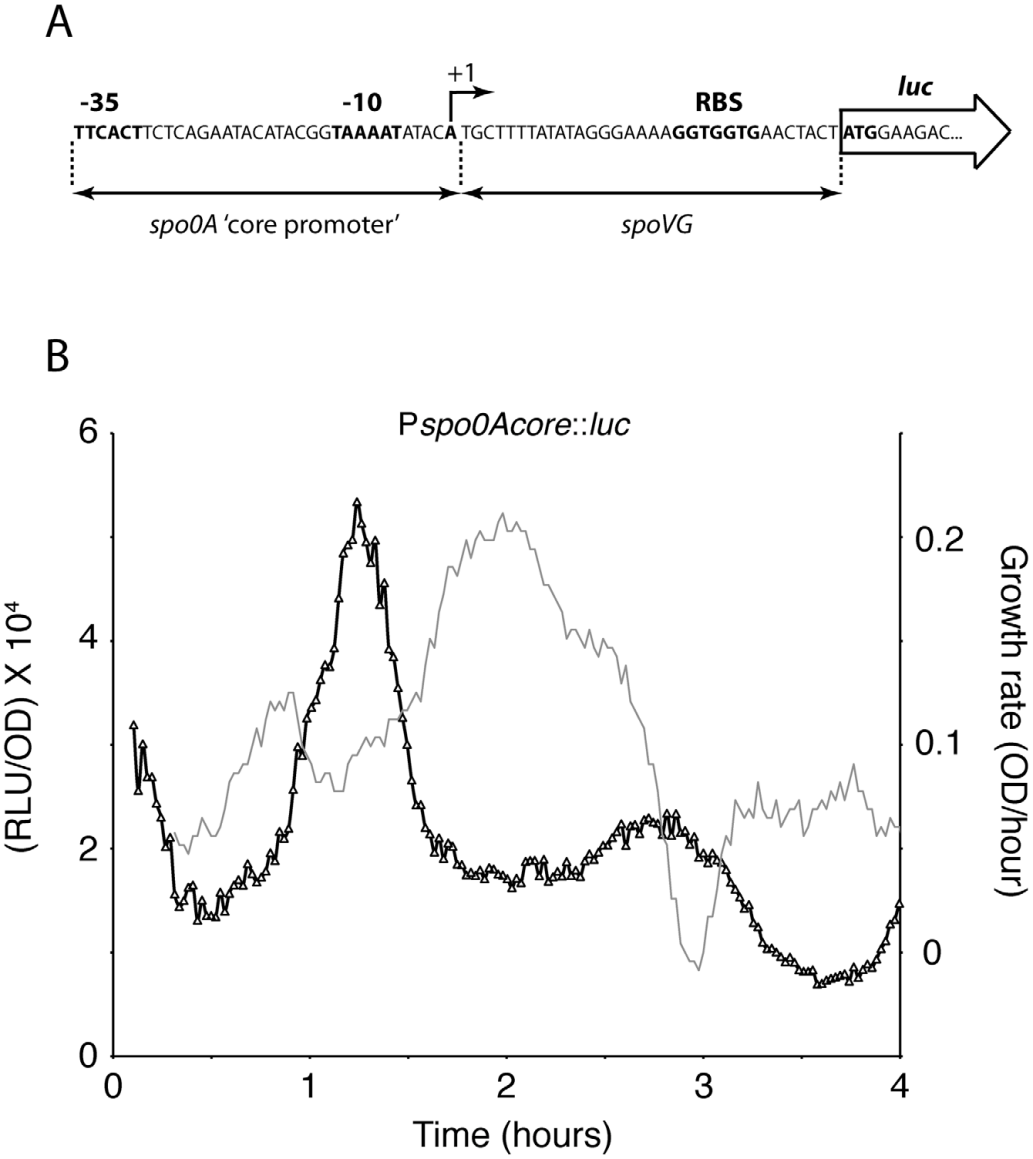
Transcription from a core *spo0A* promoter. (A) The sequence of the synthetic core promoter is shown including the start site for transcription. The ribosomal binding site sequence from *spoVG* was inserted downstream from +1. (B) A strain with P*_spo0A-core_-luc* inserted at *amyE* was grown in DSM. Light output (dark gray) and growth rate (light gray) were monitored.

The passive regulation model predicts that because the bursts in *spo0A* transcription are dependent only on the increased availability of RNA polymerase, they should be evident when expression of the *luc* reporter is driven by sequences that contain only the −35, the spacer, the −10 and the +1 sequences of the Pv promoter, presumably lacking binding sites for regulatory proteins. Such a synthetic promoter was placed in front of *luc* at the ectopic *amyE* locus (Figure 7A). The sequence downstream from +1, which includes the ribosomal binding site, was derived from an unrelated gene, *spoVG*. Figure 7B shows that the rate of expression from this promoter exhibits the same inverse relationship with growth rate as the wild-type Pv and therefore is anti-correlated with the transcription of P*rrnB*. This experiment permits a number of important conclusions. First, it excludes a role for *spo0A*-specific transcription factors in the bursts because all sequences upstream from the −35 motif and downstream from the transcription start have been removed. Second, because this construct uses the translational signals of a foreign gene (*spoVG*) it excludes a role for translational regulation. Note that these translational signals have been used in many vectors designed for use with *lacZ* as a transcriptional reporter and that *spoVG* is not known to be regulated translationally. These results provide support for the passive model.

### Effects of CodY

CodY is known to respond to the cellular pool of GTP and is clearly an important player in the response of *B. subtilis* to nutritional status [30]. Because CodY has been suggested to mediate the effects of starvation on sporulation, acting as a repressor of *spo0A*, we monitored *spo0A* transcription in a *codY* mutant (Figure 4C). Although transcription from P*spo0A* was slightly higher during stationary phase after about T_2_, the most noticeable effects of the *codY* null mutant were elimination of the fourth burst and a delay in the increased *spo0A* transcription that normally takes place after T_0_. These results suggest an unexpected positive role for CodY in the regulation of *spo0A*. During growth, pulses in *spo0A* transcription were still evident in the *codY* mutant, but with an altered pattern, although the first pulse was essentially unaffected. Comparison with the growth plot showed that the *spo0A* bursts in the *codY* strain corresponded to pauses in growth (not shown) and it is likely that these alterations in the timing of the transcription pulses in the *codY* mutant are secondary to changes in the timing of growth pauses. The complex changes exhibited by the *codY* mutant, are perhaps not surprising, because of its global effects on metabolism [50], [51].

### Effects of ppGpp synthases on *spo0A* expression

**Figure 8:**
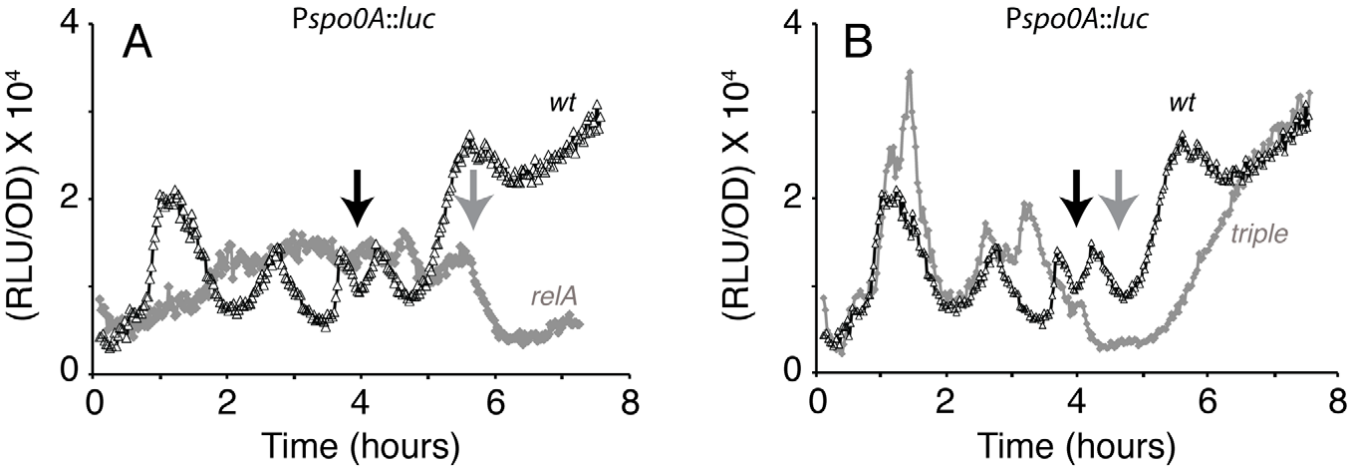
Effects of ppGpp synthase mutations on *spo0A* transcription. In both panels the black lines show *spo0A* expression in a wild-type background and the gray lines show results from mutant strains, as follows: (A) *relA* (B) *relA yjbM ywaC*.

The work of E. Freese and colleagues has shown that in response to amino acid starvation, the stringent response could regulates the onset of sporulation by depressing the GTP pool [27]. To explore the role of the stringent response on the pattern of *spo0A* transcription, we determined the effects of ppGpp synthase mutations. *B. subtilis* encodes three ppGpp synthases; RelA, YwaC and YjbM. The inactivation of *relA* alone is known to *increase* the level of ppGpp, because RelA is both a ppGpp synthase and hydrolase, while YwaC and YjbM also synthesize ppGpp but lack hydrolase activity [52]–[54]. The inactivation of *relA* alone dramatically altered the pattern of *spo0A* expression (Figure 8A). The bursts in expression during growth and the sustained increase in expression that normally occurs after T_1_ were absent and *spo0A* was expressed at a moderate level throughout the growth phase. Also, the growth rate of the mutant decreased, and growth pauses could not be detected (Figure S5B). Thus, the correlation between the pauses and the transcription bursts was maintained in this mutant, although we cannot explain how these changes result from the inactivation of *relA* and presumably from the over abundance of ppGpp. To determine the effect of the complete absence of ppGpp, we inactivated all three of the synthases by mutation. The triple mutant grew nearly as well as its wild-type parent, although it exhibited some lysis in stationary phase (Figure S5B). In this ppGpp-deficient strain, the first *spo0A* burst was relatively unaffected (Figure 8B) and was accompanied by a pause in growth similar to that of the wild type. The timing of the subsequent fluctuations in *spo0A* transcription in the triple knockout strain was altered from those of the wild-type parent. As cells entered stationary phase, the usual sustained rise was delayed relative to that of the wild-type strain. These experiments demonstrate that RelA is needed for the expression of *spo0A* that normally occurs just before and after T_0_. Individual knockouts of *yjbM* and *ywaC* had relatively minor effects on *spo0A* transcription, although the *ywaC* mutant had a depressed rate of transcription after T_2_ (Figure S5C, S5D).

Taken together, these data show that the first two bursts in *spo0A* transcription are not dependent on ppGpp, whereas the activation of transcription that accompanies entrance to the stationary phase is delayed in the ppGpp deficient mutant. This delayed rise in the absence of ppGpp was unexpected.

### CodY and the role of ppGpp in sporulation

As noted, it has been suggested that the accumulation of ppGpp in stationary phase depresses the GTP pool, which relieves repression of *spo0A* by CodY, thereby initiating spore formation [29]–[31]. We have shown that eliminating ppGpp does have a depressing effect on *spo0A* transcription early in stationary phase, while inactivation of *codY* actually appears to depress *spo0A* transcription, at least during the entry into stationary phase. Nevertheless, if the role of ppGpp in triggering sporulation were solely to inactivate CodY, the deletion of *codY* in the *relA ywaC yjbM* background should reverse the depression of *spo0A* transcription observed in the ppGpp-deficient strain and these mutations should have corresponding effects on the frequencies of spore formation (see below). The quadruple *codY relA ywaC yjbM* knockout strain was constructed and grew well. However, this strain exhibited precisely the same delayed expression of *spo0A* in stationary phase as the ppGpp-deficient parent (not shown).

To further explore the roles of ppGpp and CodY in sporulation, we determined the frequency of spore formation in mutant strains after 12, 24 and 48 hours of growth in DSM. Table 1 shows that the *codY* knockout mutant sporulated with a delay, resembling the delay it exhibits in *spo0A* transcription and reaches a final frequency of sporulation about ten-fold lower than the isogenic parent (∼2% vs 20%). The *relA* and triple *relA yjbM ywaC* mutants also exhibited delayed spore formation, and at 48 hours had about 20 and 50-fold lower sporulation frequencies than the wild-type parent, respectively. Strikingly, the quadruple *relA yjbM ywaC codY* mutant, like the *sigH* negative control, failed to form any spores, contrary to the prediction of the model. We further conclude that ppGpp plays an important role in spore formation although an alternative pathway exists and functions independently of this alarmone. Also, CodY does not function in stationary phase as a negative regulator of spore formation.

**Table 1:** Sporulation in *codY* and ppGpp-deficient mutants.

Genotype^a^	Viable count^b^	Spores/ml		
		12 hours	24 hours	48 hours
Wild-type (PP530)	0.78×10^8^	0.11×10^8^	0.12×10^8^	0.16×10^8^
Δ*sigH*	0.4×10^7^	0	0	0
Δ*codY*	3.3×10^8^	3.7×10^6^	5.8×10^6^	7.1×10^6^
Δ*relA*	1.3×10^8^	9.4×10^5^	1.1×10^6^	1.0×10^6^
Δ(*relA yjbM ywaC*)	1.0×10^8^	1.0×10^5^	2.6×10^5^	3.6×10^5^
Δ(*relA yjbM ywaC codY*)	1.5×10^8^	0	0	0

a These strains are identical to the wild-type strain (PP530) except for the indicated mutations, and are listed in the Table S1.

b The viable count after 12 hours of growth. In strains that exhibited decreased sporulation, the total viable count decreased after this point, presumably due to lysis of non-sporulating cells.

## Discussion

The data presented here demonstrate the existence of complex regulation of *spo0A* transcription operating on the vegetative promoter during the growth phase and of the sporulation promoter beginning at around T_0_. We will first consider the biological significance of the regulation of *spo0A* transcription during the growth phase and then discuss the initiation of sporulation.

### Medium-induced changes in growth and transcription

We have shown that the transcription rate of *spo0A* varies in a pulsatile manner together with the growth rate, with a pattern that is specific for different media. In each case when growth slows, a burst of transcription takes place that terminates as the growth rate increases. These changes in transcription do not reflect global changes in non-specific factors like the availability of energy or nucleotides for mRNA elongation because transcription from the P1 promoter of *rrnB* exhibits the opposite behavior (Figure 2), slowing and accelerating in concert with the growth rate. What is more, the transcription of *spoIIG* does not increase until near the stationary phase, when the concentration of Spo0A∼P has reached a level sufficient to activate its promoter, further supporting the promoter specificity of the transcriptional bursts. Although similar changes in *spo0A* transcription were recapitulated in a diauxie experiment involving starvation for glucose (Figure 3), the addition of glucose or the 20 amino acids to DSM failed to abolish the growth pauses. Many possible factors may contribute to slowing of growth in addition to exhaustion of a nutrient, such as changes in pH, depletion of oxygen and the accumulation of signaling molecules or toxic products. We suspect that the fluctuations in *spo0A* transcription are secondary to the growth pauses.

rRNA transcription in *B. subtilis* initiates with GTP and has been shown to be sensitive to decreases in the GTP pool [37]. It has been proposed that the regulatory role of the initiating nucleotide is a general one, connecting the transcription of many genes to nucleotide pool levels [47], [48]. The Pv initiating nucleotide has been mapped to the first A residue in a string of four As, helping to explain its relative independence from regulation by ppGpp (Figure 8B). When the first two As were changed to Gs, only the first burst was blunted, suggesting that the GTP pool was decreased during the corresponding pause (Figure 6B). Whatever the factors are that cause the first pause in growth, they presumably cause a drop in this pool that is independent of the stringent response (Figure 8B). Presumably the subsequent pauses in growth do not decrease the GTP pool because the A to G mutation does not affect the corresponding burst in transcription. However, our data do not imply that modulation of the NTP pools is responsible for the growth phase fluctuations in *spo0A* transcription. We suggest that the first fluctuation is caused by increased RNAP availability and that the use of several A's for initiation at Pv has evolved to prevent an *rrn*-like response when the GTP concentration drops. This suggestion rests on the assumption that an increase in *spo0A* transcription activity when growth ceases or slows down increases fitness. Below we address the nature of this fitness increase.

### The passive regulation model

We have considered two explanations for the growth-associated bursts in *spo0A* transcription. While our evidence strongly suggests that the identity of the initiating nucleotide is not responsible for the bursts, much evidence supports the idea that the bursts are caused by the decrease in stable RNA synthesis that accompanies the slowing of growth, making RNAP available for other promoters. A “passive” model based on RNAP availability has been invoked previously to explain the positive response of certain promoters to the stringent response [55], [56]. Promoters that exhibit increased activity when RNAP is made available might include low affinity constitutive promoters as well as those that are controlled by repression, because repressors usually compete with RNAP for binding. Promoters that are limited by the availability of activators would presumably not show a direct positive response to increased availability of RNAP.

Several findings are supportive of the passive model for *spo0A*. First, we find that the first two growth phase bursts in *spo0A* transcription correspond to pauses in growth. When these pauses are mitigated, as with the *relA* mutant, the bursts are also blunted (Figure 8A). When the pauses occur at different times, as with the diauxie experiment (Figure 3) or growth in other media (not shown), so do the bursts. Second, the increases and decreases in the rates of *rrnB* and *spo0A* transcription during the growth phase are anti-correlated in the wild-type background (Figure 2B). Third, we have shown that the *spo0A* and P*rrnB* bursts show the same relationship to growth pauses in the *relA ywaC yjbM* background as in the wild-type (Figure 8B and Figure S6), and most tellingly exhibit the same reciprocal relationship to one another as in the wild-type background, showing that the anti-correlations we have observed are invariant with respect to strain background. What is more, the *relA* strain, which shows little in the way of growth pauses, also shows a blunting of the transcription rate fluctuations of both P*rrnB* (Figure S6) and P*spo0A* (Figure 8A). Fourth, a core *spo0A* promoter, stripped of potential protein binding sites exhibits the same bursts, again reciprocally related to increases and decreases in the growth rate. Fifth, similar bursts were noted for *abrB* in both the presence and absence of *spo0A* (Figure 1B) and for *mecA* (not shown) but were not observed for *spoIIG* (Figure 1A). These observations are precisely what is predicted by the passive model, because *abrB* is normally repressed by both Spo0A and AbrB, *mecA* is constitutively expressed [57] and *spoIIG* is activated by high concentrations of Spo0A∼P. Fluctuations were also absent for several other activator-driven promoters unrelated to sporulation, in a medium in which *spo0A* still exhibits fluctuations correlated with growth pauses (not shown). Finally, and perhaps most tellingly, because it is known that stable RNA synthesis engages a major fraction of RNAP during rapid growth [46] and that the activities of many promoters are limited by the availability of RNAP, it seems almost inevitable that a growth slowdown will cause an uptick in transcription of these promoters.

As implied above, we believe it likely that the passive mechanism is not an accident of evolution, but that the mode of regulation of genes (repression, activation, constitutivity, affinity for RNA polymerase) is selected *in part* to produce an appropriate response to growth slowdown.

### The initiation of spore formation in stationary phase

The *sigH* mutant decreased the stationary phase transcription of *spo0A*, reflecting the well-documented phenomenon of promoter switching [11], [12]. The absence of Spo0A had a depressing effect on *spo0A* transcription after T_0_, probably due to the inducing effect of Spo0A∼P on *sigH* as well as the direct positive effect of Spo0A∼P on its own promoter [39], [40]. These data are consistent with the emerging conclusion that the stationary phase increase in *spo0A* transcription is secondary to increased flow of phosphoryl groups through the phosphorelay [7], [10]. The pattern of *spo0A* transcription at T_0_ and in early stationary phase is characteristic and complex, exhibiting waves and stepwise increases. The third wave, which occurs at T_0_, is somewhat affected by the *sigH* mutant (Figure 4A) but is also accompanied by a pause in the growth rate (Figure 1A). It appears to exhibit characteristics of both growth phase and early stationary phase regulation. We do not understand the allover pattern of *spo0A* transcription in stationary phase in detail, but it hints at a complex and programmed regulation of the phosphorelay with consequent changes in the transcription of *spo0A*. This pattern would not be detectable using the traditional reporter technologies.

The ppGpp-deficient (*relA ywaC yjbM*) strain shows a delay in the rise in the stationary phase induction of *spo0A* transcription. The *relA* mutant, which is expected to accumulate excess ppGpp, shows a more extreme effect, perhaps with a delayed rise beginning at about T_2_ (Figure 8A) suggesting that excess ppGpp is inhibitory and that this molecule may function optimally in sporulation within a narrow concentration range. The triple ppGpp deficient mutant shows a delay in sporulation, and even after 48 hours a 50-fold lower sporulation frequency than the wild-type strain. When considering the role of ppGpp in sporulation, it is interesting that an effect of this molecule on the activation of SigH has been suggested [58], which could explain the decreased transcription of *spo0A* in the *ywaC yjbM relA* mutant at a time when Ps is normally activated. It is also possible that the accumulation of ppGpp or the consequent decrease in GTP as cells enter stationary phase somehow activates the phosphorelay. Clearly the proper regulation of ppGpp synthesis/hydrolysis is important for the optimal initiation of sporulation, although a backup mechanism exists that is ppGpp independent (Table 1).

It has been proposed that the stringent response depresses the GTP pool, reversing repression of *spo0A* by CodY as cells initiate sporulation [29]–[31]. Also, Piggot and Hilbert [59] have suggested, based on transcriptional profiling data [50], that CodY represses the transcription of *kinB*, *phrE* and *phrA*, and reversal of this repression might increase the concentration of Spo0A∼P. However, we have found that the inactivation of *codY* has a depressing effect on both *spo0A* induction and on spore formation, suggesting a positive role for CodY. Perhaps CodY represses a gene that is initially inhibitory for spore formation such as *rapA*, which encodes a Spo0F∼P phosphatase [50]. Inactivation of *codY* in the ppGpp-deficient triple mutant background did not restore the expression of *spo0A*; in fact the *codY relA ywaC yjbM* quadruple mutant failed to sporulate at all, although it grew well (Table 1). It appears that the simple model proposed previously is unlikely to be true. The inactivation of *codY* does permit spore formation to occur in rich media in which spores do not normally form [30]. A main task of CodY may be to prevent inappropriate sporulation in rich media. Clearly, activation of sporulation and of *spo0A* transcription by ppGpp is complex and involves pathways in addition to repression by CodY.

### Two modes of transition for development

The growth phase pulses in *spo0A* transcription result in the production of Spo0A∼P (Figure 1B). We suggest that the induction of *spo0A* transcription in response to decreased growth rate, primes the average cell for entry into a developmental pathway. We have shown indeed that rare transitions to competence increase in frequency following these pulses. This may be a bet-hedging strategy to insure that some genomes will survive even when faced with sudden overwhelming adversity. It has been shown that the programmed increase in the probability of transition that occurs in some media at about T_0_ depends on a spike in “basal” *comK* transcription. Here we show that spikes in *spo0A* transcription cause just such burst in the expression of *comK* even during exponential growth in DSM (Figure 5). We propose that spikes in *spo0A* transcription may also increase the probability of transition to other developmental pathways, such as the formation of biofilms, by placing a few cells near the transition threshold. Sporulation may not fit this paradigm because the Spo0A∼P threshold for this pathway may be too high. If the stress is removed in time and growth resumes, the rate of *spo0A* transcription will decrease and the accumulation of Spo0A and hence of Spo0A∼P will therefore also decrease and the transition probability would be expected to return to a very low basal level.

Our studies therefore point to the existence of two types of developmental regulation, both dependent on *spo0A*; an early alarm system and a later more sustained response. In the first mode, rare cells escape the controls imposed on development when growth slows in response to stress. In the second mode, programmed changes in signal transduction pathways ensure that a major fraction of cells escape these controls. Although the sporulation and competence pathways both rely on Spo0A∼P, the development of spores in stationary phase depends on increased flux through the phosphorelay [7], [10]. Increases in *spo0A* transcription are apparently secondary to this flux increase, responding in what has been described as a “just-in-time mechanism” [7]. In contrast, during the bet-hedging mode for competence that we have described in this study, transcription from Pv is limiting, perhaps responding passively to the increased availability of RNAP. It remains to be seen whether changes in *spo0A* transcription likewise define the window of opportunity for competence as cells enter stationary phase.

## Materials and Methods

### Strains and strain construction

*Bacillus subtilis* strains were constructed by transformation into BD630 (*his leu met*), and all the strains were therefore isogenic. The details of strain and plasmid constructions are respectively presented in the Tables S1 and S2. When it was desired to combine the constructs described below, this was performed by transformation, with selection for the appropriate antibiotic resistance marker. For transformation, competent cultures were prepared and incubated in competence medium with transforming DNA (1 µg/ml) for 30 min at 37°C [36]. The strains are listed in Table S1. The minimal medium for the diauxie experiment was S7, supplemented with trace elements [60], [61]. Most growth experiments were carried out in DSM [34].

### Luciferase assay

For the detection of luciferase activity, strains were first grown in LB medium to an optical density at 600 nm (OD_600_) of 2. Cells were then centrifuged and resuspended in fresh DSM, adjusting all the cultures to an OD_600_ of 2. These pre-cultures were then diluted 20 fold in fresh DSM and 200 µl was distributed in each of two wells in a 96-well black plate (Corning). 10 µl of luciferin were added to each well to reach a final concentration of 1.5 mg/ml (4.7 mM). The cultures were incubated at 37°C with agitation in a PerkinElmer Envision 2104 Multilabel Reader equipped with an enhanced sensitivity photomultiplier for luminometry. The temperature of the clear plastic lid was maintained at 38°C to avoid condensation. Relative Luminescence Unit (RLU) and OD_600_ were measured at 1.5 min intervals. Further details are presented in Text S1.

### Construction of deletions

To inactivate *B. subtilis* genes, we replaced them cleanly with antibiotic cassettes without using a vector. This method was employed for the knockout of the *yjbM*, *ywaC*, *codY* and *sigH* genes. All the PCR primers used in this work are listed in the Table S2. We first amplified 1 kb fragments upstream and downstream of the gene. These fragments are each flanked with one restriction site at the junction with respectively the ‘start’ or the ‘stop’ of the gene. In parallel, we amplified an antibiotic cassette flanked with the corresponding restriction sites. The three fragments were then digested and ligated together. The ligated DNA was then purified through a QIAquick column. The desired product, corresponding to ligation of the three fragments, was purified from an agarose gel. The purified band was then amplified by PCR using the outside primers previously used to amplify the upstream and downstream fragments. After further purification on QIAquick columns, the full fragment (upstream + antibiotic cassette + downstream) was used to transform *B. subtilis*, yielding a double crossover event between the chromosome and the region of homology, replacing the gene with the antibiotic cassette.

### Construction of Pv mutation

Mutagenesis of the *spo0A* promoter in the plasmid pUC18cm-P*_spo0A_*::luc was carried out using the ‘Change-IT Multiple Mutation Site Directed Mutagenesis’ kit from USB, with the primer A→G (1+2) listed in Table S2. Once verified by sequencing, the mutated plasmids were integrated by Campbell-like recombination and the structure of the integration event was verified by sequencing a relevant PCR fragment from the chromosome.

### Construction of luciferase promoter fusion strains

A 1 Kb fragment ending with the initiating codon of the gene of interest, and containing the promoter, was amplified by PCR from the *B. subtilis* chromosome. A single nucleotide was inserted in the primer to restore the correct reading frame. Primers are listed in Table S2. This fragment was cut by *Kpn*I/*Nco*I in sites present at the extremities of the primers used for the amplification. In parallel, the luciferase gene was cut from the pGL3 plasmid (Promega) by *Nco*I/*Bam*H1 digestion. A three-fragment ligation was then carried out between the promoter of interest, the luciferase gene and plasmid pUC18Cm digested with *Kpn*I and *Bam*H1. The resulting plasmid, pCU18cm-promoter::luc, which cannot replicate autonomously in *B. subtilis*, was used to transform *B. subtilis* where it integrated, by single crossover. This event reconstructs the “normal” regulatory region in front of the fusion and a complete copy of the gene of interest, downstream of the fusion.

### Construction of the *spo0A* core promoter fusion

The *luciferase* gene was amplified from the plasmid pGL3 (Promega) using the primers ‘OAcorepromo’ and ‘luc2’ (see Table S2). The ‘OAcorepromo’ primer allowed us to add the *spo0A* core promoter and the *spoVG*'s RBS sequence upstream of the *luc* gene. The PCR product was cut by *Bam*H*1*/*Eco*RI and cloned in the plasmid pDR111 digested with the same enzymes. The resulting plasmid, pDR111-P*spo0Acore*::*luc*, was used to transform *B. subtilis* where it integrated, by double crossover, at the *amyE* locus.

### Spore counts

We incubated a single colony of each strain in 10 ml of DSM at 37°C with shaking. After 12, 24 and 48 hours, samples were taken and serial dilutions were made and plated on LB agar before and after heating for 10 min at 80°C.

### Microscopy

From cultures of BD5486 and BD5487 growing in the plate reader in DSM, samples were taken after 0.65 and 3.4 hours, before and after the two major bursts in *spo0A* transcription. Aliquots (1 ml) of each sample were fixed by the addition of 20 µl of Na_2_HPO_4_, pH 7.4 and 100 µl of formaldehyde. The samples were kept on ice for one hour and then washed twice in PBS. One µl of each treated sample was placed on a 1% agarose pad. All images were acquired using Volocity v 4.1 (Perkin Elmer) and a Nikon 90i fluorescence microscope with filters appropriate for detection of GFP.

### mRNA extraction and *spo0A* transcription start mapping

To obtain freshly growing cells from the strain PP530 (P*spo0A*::*luc*), overnight cultures grown at 30°C in LB were diluted 50-fold into fresh DSM. At T_−1_ and T_3_, 15-ml samples were taken, rapidly chilled, pelleted by centrifugation for 10 min at 4°C, and resuspended in 1 ml RNApro solution (MPbio). RNA was then extracted using FastRNA Pro Blue Kit (MPbio). 5′ RACE-PCR was carried out using the 5′ Race System for Rapid Amplification of cDNA Ends Kit (Invitrogen). Sequences of gene-specific primers (GSP1-0A and GSP2-0A) used for the mapping are shown in Table S2. The final PCR products obtained with the Race Kit were separated by gel electrophoresis, purified, cloned into the *BamH*I and *Sal*I sites of pUC18 and sequenced, using the primer seq+1-0A (Table S2).

## Supporting Information

Figure S1 Properties of the luciferase assay. All of these curves were obtained with the P*spo0A-luciferase* fusion. (A) Maximum light output at the fifth burst (solid line) and the first burst (dashed line) plotted as a function of the initial luciferin concentration in the growth medium. (B) Luciferin was added to 1.5 mg/ml at the times indicated by the vertical arrows. (C) Puromycin (200 µg/ml) was added to one of duplicate cultures at the time indicated by the arrow. (D) The data from panel C, plotted with an expanded time scale.
(TIF)

Figure S2 Reproducibility of the luciferase assay. The results of duplicate samples of the strain PP530 (P*spo0A*::*luc*) from two independent experiments are shown. The duplicate growth curves and light output curves from each experiment are shown in shades of red and blue.
(TIF)

Figure S3 Correlation between *spo0A* transcription and the concentration of Spo0A protein. Strain PP530 was used for this experiment. Panels (A) and (B) show results in S7 medium with the indicated concentration of Glucose and Arabinose (Figure 3B) and in DSM, respectively. Samples were taken from the plate reader for Western blotting using anti-Spo0A antiserum at the times indicated by the red arrows in the figure. Equal amount of total protein were loaded on each lane of the gels. The antiserum was a kind gift from M. Fujita.
(TIF)

Figure S4 Effects of gene inactivations known to affect *spo0A* expression. In each panel, light output from a wild-type strain (dark lines) is compared to that from an isogenic mutant (gray lines). (A) Δ*kinA* (B) Δ*kinB* (C) Δ*kinC* (D) Δ*kinD* (E) Δ*kinE* (F) Δ*spo0J* (G) Δ(*soj spo0J*) (H) Δ*epsH* (as a control for panel I) (I) Δ*epsH* Δ*sinR*. The downward-facing arrows show T_0_ for the wild type (black) and mutant (gray) strains.
(TIF)

Figure S5 Effects of mutations in ppGpp synthases on growth and transcription of *spo0A*. (A) Growth curves of strains with deletions of *yjbM* (green), *ywaC* (purple), *ywaC* and *yjbM* (blue) and the wild type strain (black). (B) Growth curves of strains with deletions of *relA* (red), *relA* and *yjbM* (green), *ywaC* and *relA* (purple), *relA*, *ywaC* and *yjbM* (blue), and the wild type strain (black). (C) Transcription of *spo0A* in wild type (black) and *yjbM* (gray) backgrounds. (D) Transcription of *spo0A* in wild type (black) and *ywaC* (gray) backgrounds. (E) Transcription of *spo0A* in wild type (black) and *ywaC yjbM* (gray) backgrounds. (F) Transcription of *spo0A* in wild type (black) and *relA* (gray) backgrounds. (G) Transcription of *spo0A* in wild type (black) and *relA yjbM* (gray) backgrounds. (H) Transcription of *spo0A* in wild type (black) and *relA ywaC* (gray) backgrounds.
(TIF)

Figure S6 Effects of ppGpp synthase mutations on *rrnB* transcription. In both panels the black lines show *rrnB* expression in a wild-type background and the gray lines show results from mutant strains, as follows: (A) *relA* (B) *relA yjbM ywaC*.
(TIF)

Table S1 Bacterial strains used for this study.
(RTF)

Table S2 Sequence of oligonucleotide primers used.
(RTF)

Text S1 The method that allows us to measure the *rate* of transcription of a given gene during the growth of *B. subtilis* using the Firefly luciferase as a reporter gene is described in detail.
(RTF)
