# Supplementary figures and images for: Fluctuations in spo0A Transcription Control Rare Developmental Transitions in Bacillus subtilis
Source: PLoS Genet. 2011 Apr 28;7(4):e1002048. doi: 10.1371/journal.pgen.1002048 (PMC3084206; doi:10.1371/journal.pgen.1002048)

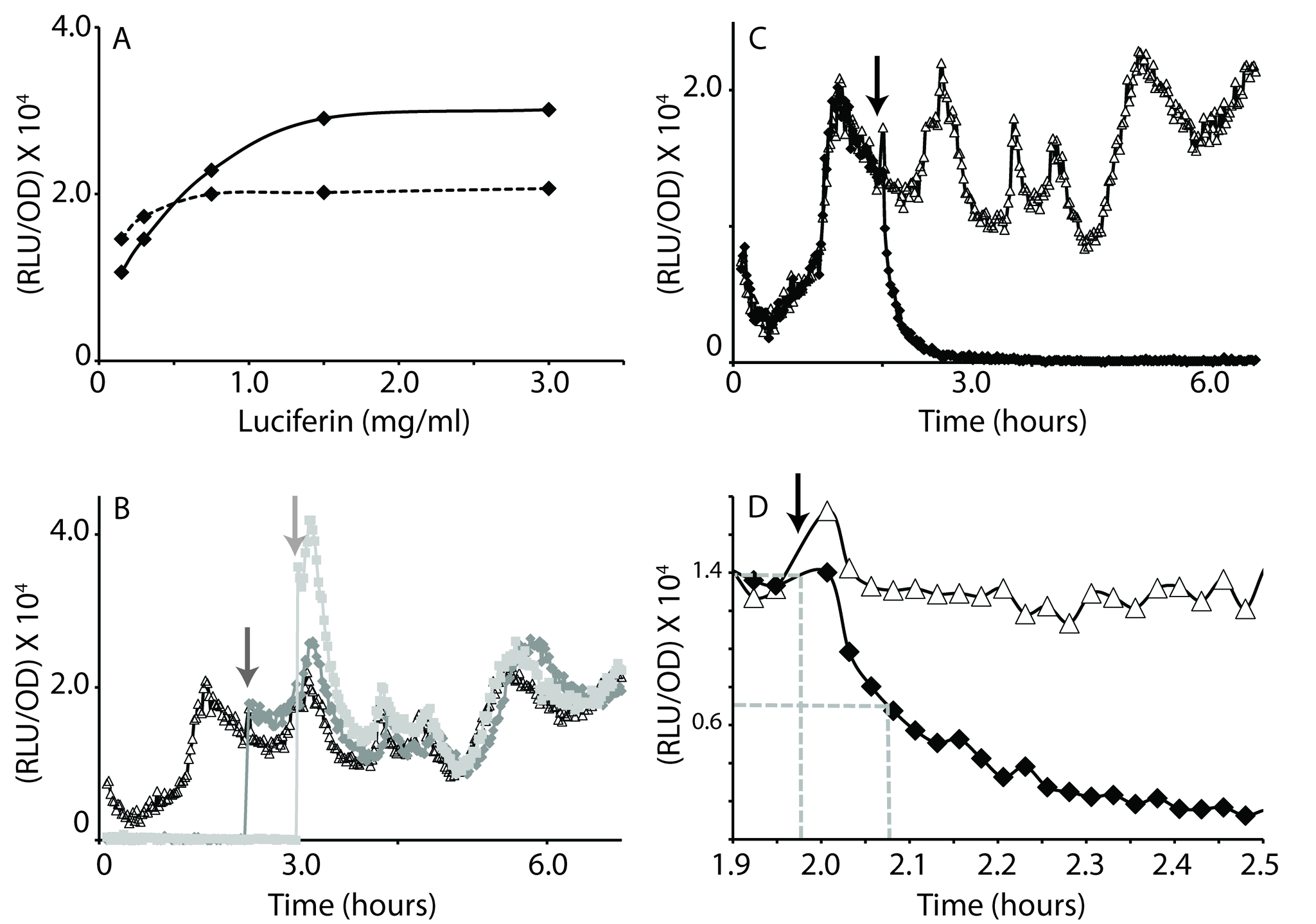

Supplement: Figure S1 — Properties of the luciferase assay. All of these curves were obtained with the Pspo0A-luciferase fusion. (A) Maximum light output at the fifth burst (solid line) and the first burst (dashed line) plotted as a function of the initial luciferin concentration in the growth medium. (B) Luciferin was added to 1.5 mg/ml at the times indicated by the vertical arrows. (C) Puromycin (200 µg/ml) was added to one of duplicate cultures at the time indicated by the arrow. (D) The data from panel C, plotted with an expanded time scale. (TIF) [file pgen.1002048.s001.tif]

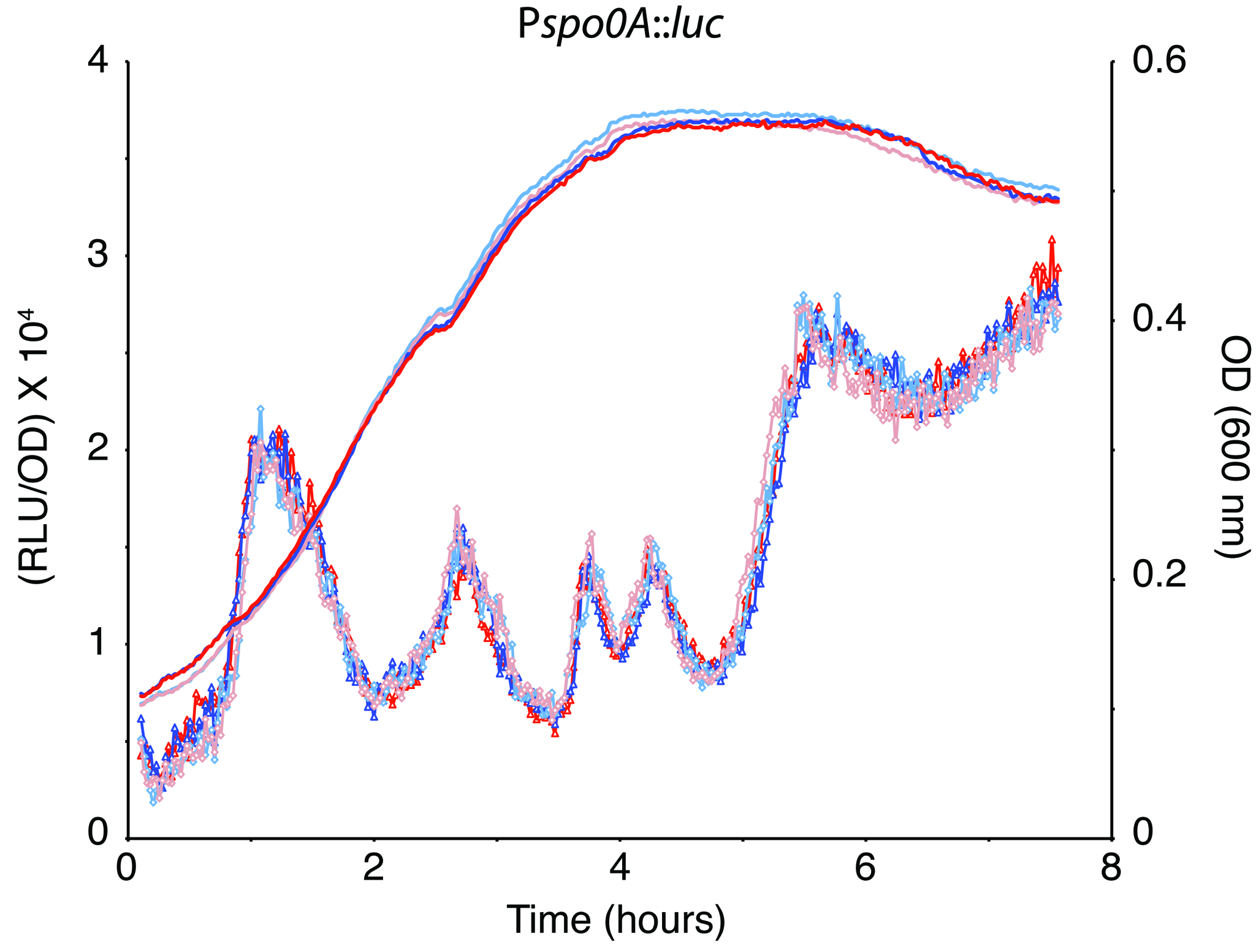

Supplement: Figure S2 — Reproducibility of the luciferase assay. The results of duplicate samples of the strain PP530 (Pspo0A::luc) from two independent experiments are shown. The duplicate growth curves and light output curves from each experiment are shown in shades of red and blue. (TIF) [file pgen.1002048.s002.tif]

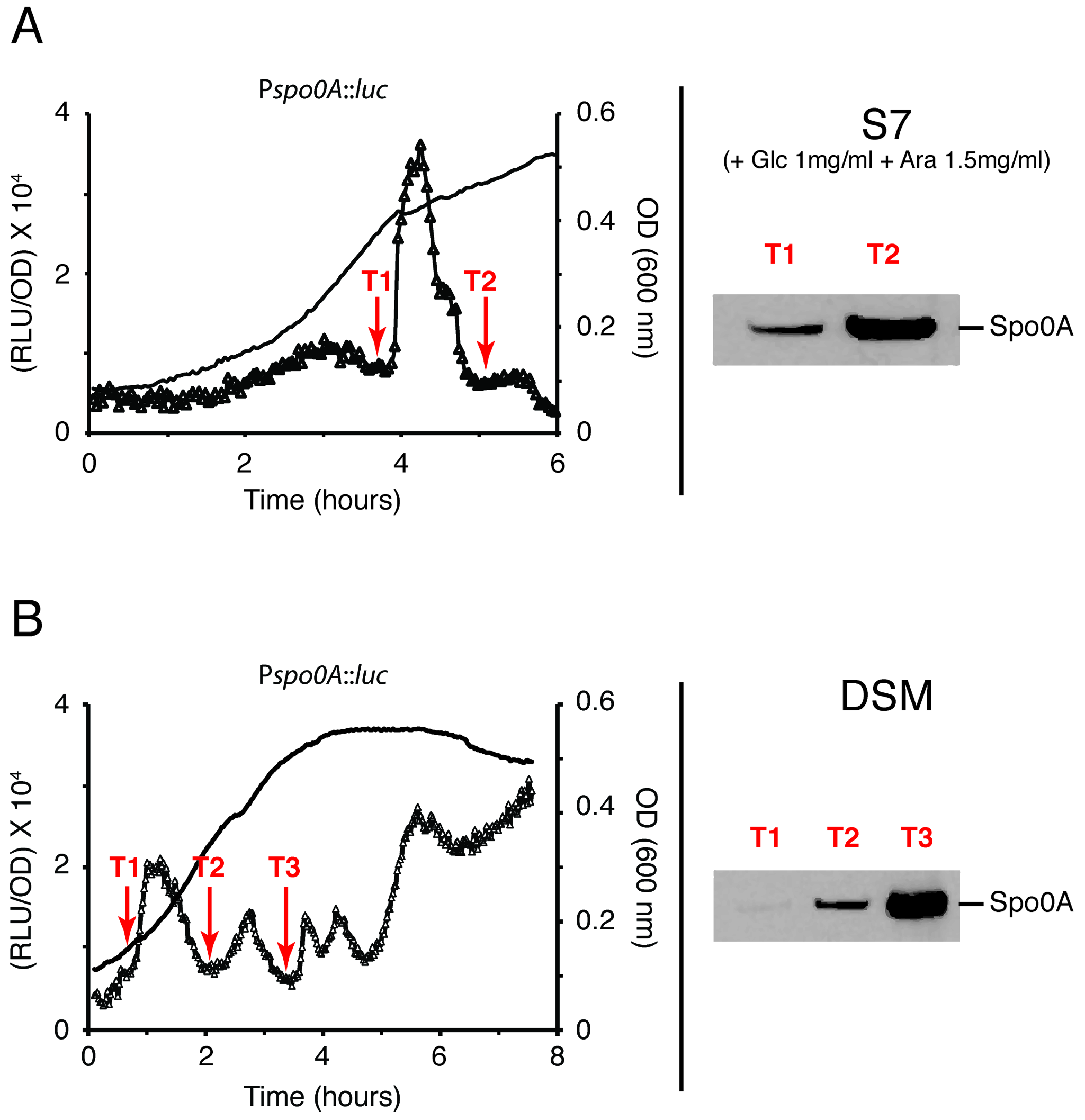

Supplement: Figure S3 — Correlation between spo0A transcription and the concentration of Spo0A protein. Strain PP530 was used for this experiment. Panels (A) and (B) show results in S7 medium with the indicated concentration of Glucose and Arabinose (Figure 3B) and in DSM, respectively. Samples were taken from the plate reader for Western blotting using anti-Spo0A antiserum at the times indicated by the red arrows in the figure. Equal amount of total protein were loaded on each lane of the gels. The antiserum was a kind gift from M. Fujita. (TIF) [file pgen.1002048.s003.tif]

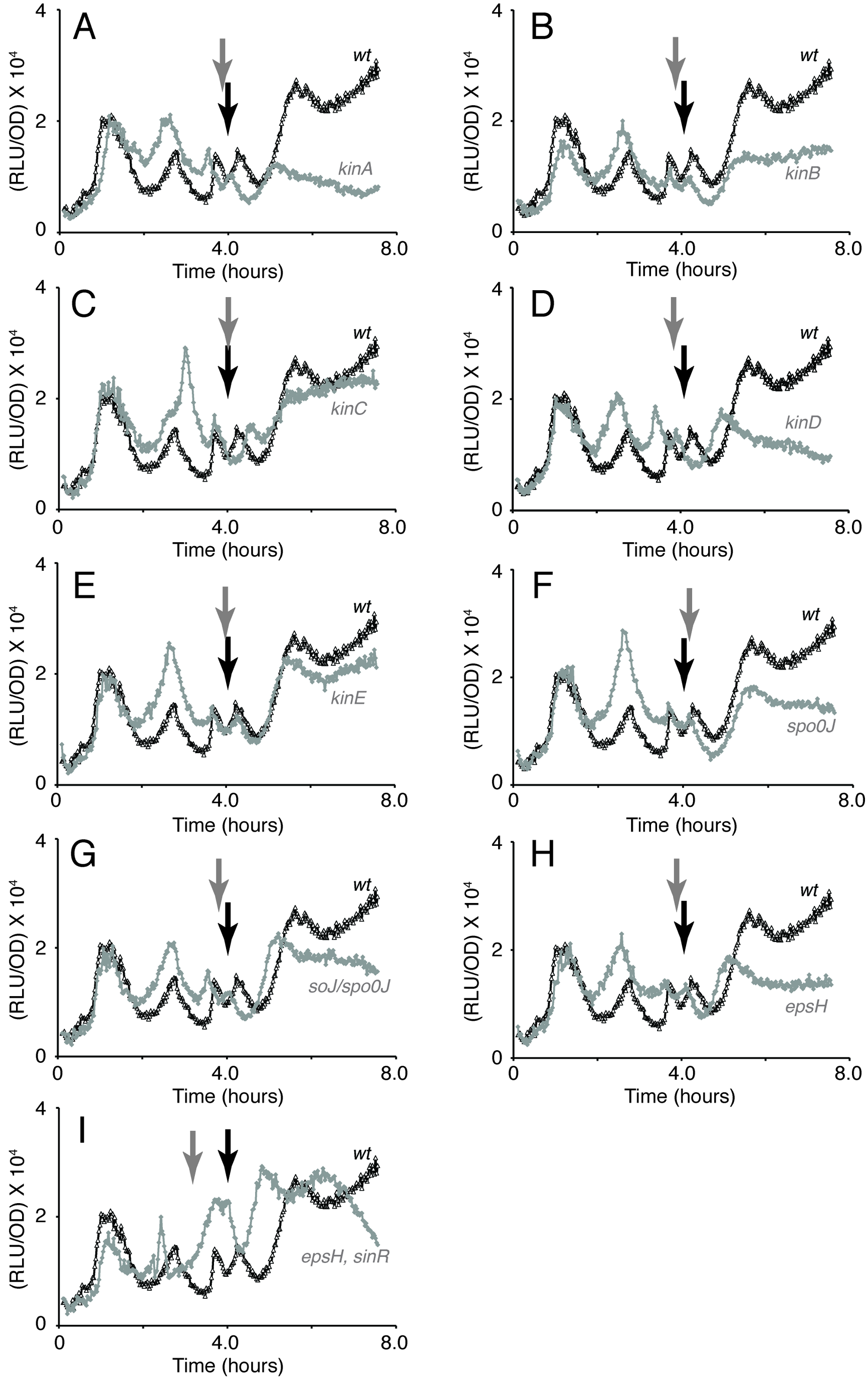

Supplement: Figure S4 — Effects of gene inactivations known to affect spo0A expression. In each panel, light output from a wild-type strain (dark lines) is compared to that from an isogenic mutant (gray lines). (A) ΔkinA (B) ΔkinB (C) ΔkinC (D) ΔkinD (E) ΔkinE (F) Δspo0J (G) Δ(soj spo0J) (H) ΔepsH (as a control for panel I) (I) ΔepsH ΔsinR. The downward-facing arrows show T0 for the wild type (black) and mutant (gray) strains. (TIF) [file pgen.1002048.s004.tif]

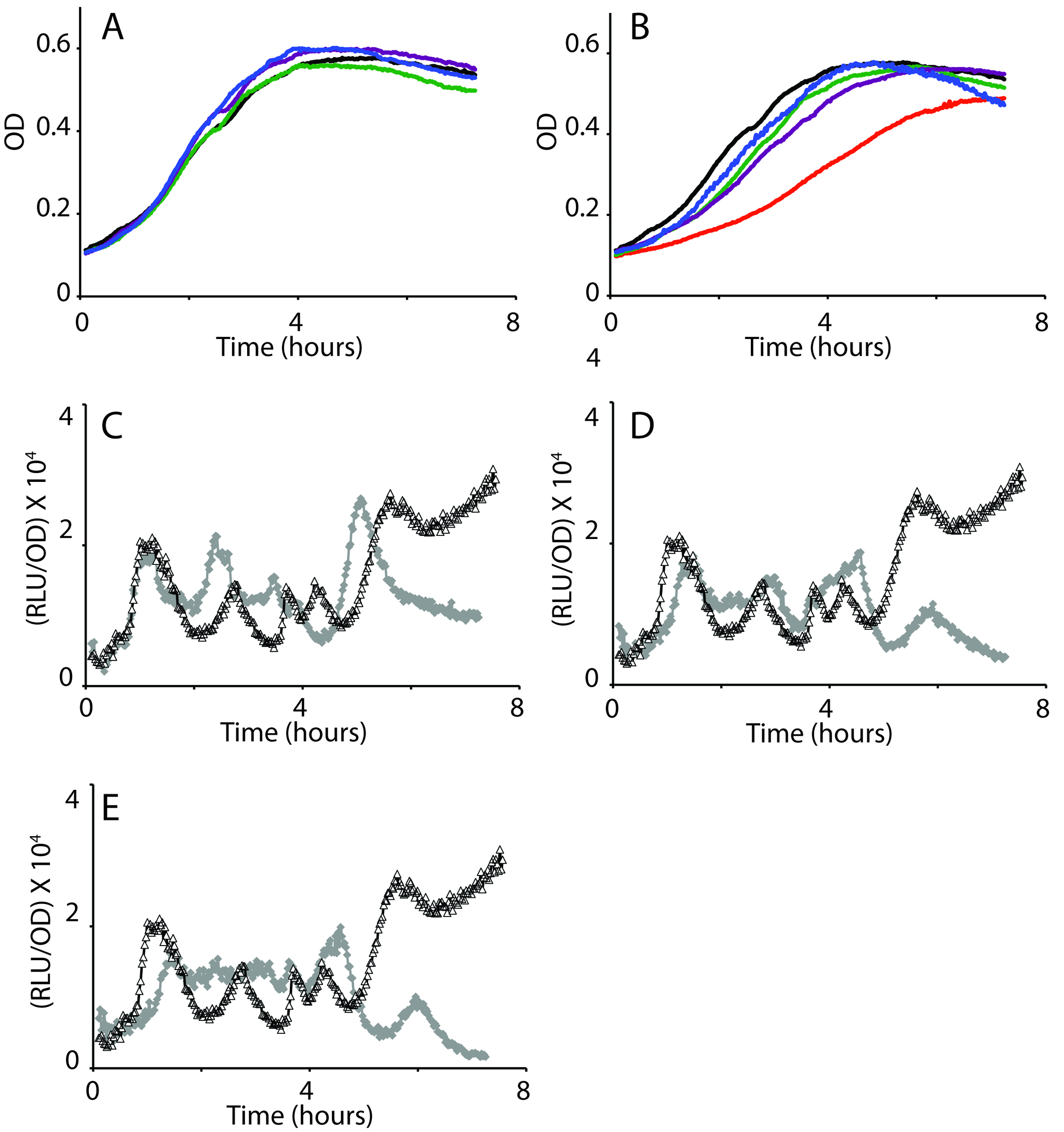

Supplement: Figure S5 — Effects of mutations in ppGpp synthases on growth and transcription of spo0A. (A) Growth curves of strains with deletions of yjbM (green), ywaC (purple), ywaC and yjbM (blue) and the wild type strain (black). (B) Growth curves of strains with deletions of relA (red), relA and yjbM (green), ywaC and relA (purple), relA, ywaC and yjbM (blue), and the wild type strain (black). (C) Transcription of spo0A in wild type (black) and yjbM (gray) backgrounds. (D) Transcription of spo0A in wild type (black) and ywaC (gray) backgrounds. (E) Transcription of spo0A in wild type (black) and ywaC yjbM (gray) backgrounds. (F) Transcription of spo0A in wild type (black) and relA (gray) backgrounds. (G) Transcription of spo0A in wild type (black) and relA yjbM (gray) backgrounds. (H) Transcription of spo0A in wild type (black) and relA ywaC (gray) backgrounds. (TIF) [file pgen.1002048.s005.tif]

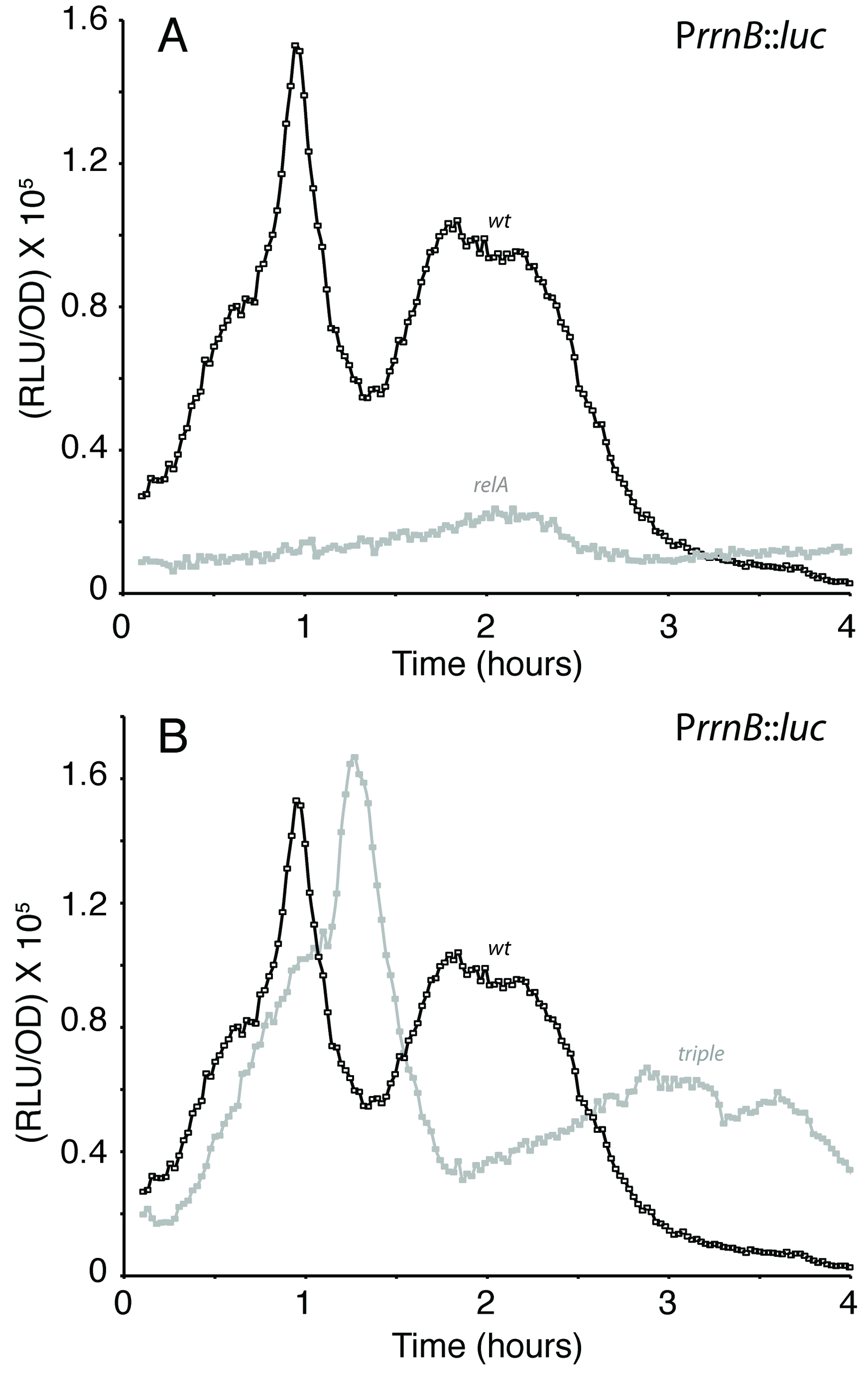

Supplement: Figure S6 — Effects of ppGpp synthase mutations on rrnB transcription. In both panels the black lines show rrnB expression in a wild-type background and the gray lines show results from mutant strains, as follows: (A) relA (B) relA yjbM ywaC. (TIF) [file pgen.1002048.s006.tif]
