# Supplementary material for: Fluctuations in spo0A Transcription Control Rare Developmental Transitions in Bacillus subtilis
Source: PLoS Genet. 2011 Apr 28;7(4):e1002048. doi: 10.1371/journal.pgen.1002048 (PMC3084206; doi:10.1371/journal.pgen.1002048)
Supplement: Table S1 — Bacterial strains used for this study. (RTF) [file pgen.1002048.s007.rtf]

Fluctuations in spo0A transcription control rare developmental transitions in Bacillus subtilis:
Table S1

Table S1. Bacterial strains used for this study
B. subtilis strains	Genotype1	Source2	
BD630	his leu-8 metB5	Lab strain	
PP533	his leu-8 metB5, PspoIIG-luc cm	This study	
BD5246	his leu-8 metB5, PabrB-luc cm	This study	
BD5382	his leu-8 metB5, PabrB-luc cm, Δspo0A::kan	This study	
BD5277	his leu-8 metB5, PrrnB-luc cm	This study	
BD5289	his leu-8 metB5, PrrnB-luc cm, ΔrelA::ery	This study	
BD5401	his leu-8 metB5, PrrnB-luc cm, ΔrelA::ery,  ΔyjbM::tet, ΔywaC::kan, ΔcodY::spc	This study	
PP530	his leu-8 metB5, Pspo0A-luc cm	This study	
BD5015	his leu-8 metB5, Pspo0A-luc cm, ΔkinA::ery	This study	
BD4998	his leu-8 metB5, Pspo0A-luc cm, ΔkinB::phleo	This study	
BD4999	his leu-8 metB5, Pspo0A-luc cm, ΔkinC::kan	This study	
BD5000	his leu-8 metB5, Pspo0A-luc cm, ΔkinD::tet	This study	
BD5001	his leu-8 metB5, Pspo0A-luc cm, ΔkinE::tet	This study	
BD5002	his leu-8 metB5, Pspo0A-luc cm, Δspo0A::kan	This study	
BD5003	his leu-8 metB5, Pspo0A-luc cm, Δspo0H::tet	This study	
BD5120	his leu-8 metB5, Pspo0A-luc cm, Δspo0J::spc	This study	
BD5121	his leu-8 metB5, Pspo0A-luc cm, ΔsoJ/spo0J::spc	This study	
BD5122	his leu-8 metB5, Pspo0A-luc cm, ΔepsH::tet	This study	
BD5123	his leu-8 metB5, Pspo0A-luc cm, ΔepsH::tet, ΔsinR::phleo	This study	
BD5124	his leu-8 metB5, Pspo0A-luc cm, ΔcodY::spc	This study	
BD5239	his leu-8 metB5, Pspo0A-luc cm, ΔrelA::ery	This study	
BD5390	his leu-8 metB5, Pspo0A-luc cm, ΔyjbM::tet	This study	
BD5391	his leu-8 metB5, Pspo0A-luc cm, ΔywaC::kan	This study	
BD5392	his leu-8 metB5, Pspo0A-luc cm, ΔyjbM::tet, ΔywaC::kan	This study	
BD5393	his leu-8 metB5, Pspo0A-luc cm, ΔrelA::ery,  ΔyjbM::tet	This study	
BD5394	his leu-8 metB5, Pspo0A-luc cm, ΔrelA::ery,  ΔywaC::kan	This study	
BD5395	his leu-8 metB5, Pspo0A-luc cm, ΔrelA::ery,  ΔyjbM::tet, ΔywaC::kan	This study	
BD5467	his leu-8 metB5, Pspo0A-luc cm, ΔrelA::ery,  ΔyjbM::tet, ΔywaC::kan, ΔcodY::spc	This study	
BD5241	his leu-8 metB5, Pspo0A-luc cm, ΔrelA::ery,  aprE::Pspac-relAD264G	This study	
BD5386	his leu-8 metB5, Pspo0A-luc (A->G 1+2) cm	This study	
BD5486	his leu-8 metB5, Pspo0A-luc cm, PcomK-gfp::tet	This study	
BD5487	his leu-8 metB5, Pspo0A-luc cm, Δspo0A::kan, PcomK-gfp::tet	This study	
BD5677	his leu-8 metB5, amyE::Pspo0Acore-luc spc	This study	

1All of the luc fusions were integrated at the native loci by single reciprocal (Campbell-like) recombination, preserving the wild-type native locus and placing the fusion under control of all the upstream regulatory sequences.
2The sources of the knockout mutations, other than ywAC and yjbM are as follows: Δspo0A (M. Fujita), ΔkinA,  ΔkinB, and  ΔkinC [1], ΔkinD and  ΔkinE (R. Losick),  Δspo0H [2], Δspo0J and Δsoj [3]  ΔsinR [4], ΔepsH [5], ΔcodY (L. Sonenshein), ΔrelA and relAD264G [6].

References
1. Quisel JD, Burkholder WF, Grossman AD (2001) In vivo effects of sporulation kinases on mutant Spo0A proteins in Bacillus subtilis. J Bacteriol 183: 6573-6578.
2. Dubnau E, Ramakrishna N, Cabane K, Smith I (1981) Cloning of an early sporulation gene in Bacillus subtilis. J Bacteriol 147: 622-632.
3. Quisel JD, Grossman AD (2000) Control of sporulation gene expression in Bacillus subtilis by the chromosome partitioning proteins Soj (ParA) and Spo0J (ParB). J Bacteriol 182: 3446-3451.
4. Mandic-Mulec I, Gaur N, Bai U, Smith I (1992) Sin, a stage-specific repressor of cellular differentiation. J Bacteriol 174: 3561-3569.
5. Branda SS, Gonzalez-Pastor JE, Ben-Yehuda S, Losick R, Kolter R (2001) Fruiting body formation by Bacillus subtilis. Proc Natl Acad Sci U S A 98: 11621-11626.
6. Nanamiya H, Kasai K, Nozawa A, Yun CS, Narisawa T, et al. (2007) Identification and functional analysis of novel (p)ppGpp synthetase genes in Bacillus subtilis. Mol Microbiol 67: 291-304.
