# Supplementary material for: Fluctuations in spo0A Transcription Control Rare Developmental Transitions in Bacillus subtilis
Source: PLoS Genet. 2011 Apr 28;7(4):e1002048. doi: 10.1371/journal.pgen.1002048 (PMC3084206; doi:10.1371/journal.pgen.1002048)
Supplement: Table S2 — Sequence of oligonucleotide primers used. (RTF) [file pgen.1002048.s008.rtf]

Fluctuations in spo0A transcription control rare developmental transitions in Bacillus subtilis:
Table S2

Table S2. Sequence of oligonucleotide primers used
Primer	Sequence	
spo0A1	5'- GCA GCA GGT ACC TAA ACA GAA AAT CAA AAC GAA GCT GAT CCC AGA -3'	
spo0A2	5'- G CAG CAC CAT GGC CAC GTT TCT TCC TCC CCA AAT GTA GTT AAC AGG ATT CAC -3'		
spoIIGA1	5'- GCA GCA GGT ACC  ATA CAC AAA TAA GGA TAG CGT CAC TGT AAA AGG AAA CGC TTC -3'	
spoIIGA2	5'- G CAG CAC CAT GGC CAC ATC TGA CTC CTT TCT TTC TTG CCT CAC GCT GTT CCC -3'	
abrB1	5'- GCA GCA GGT ACC TGA TCA ATT CAA CTG GGT GAC CGC TGT -3'	
abrB2	5'- G CAG CAC CAT GGC CAT TCT CCT CCC AAG AGA TAC TTA TTT GTT TAA ATT ATA TTT TTC TTC GTC ATT ATT CG -3'	
rrnB1	5'- CGA CGA GGT ACC TGT TTT TGA TAC AGC GGC TTT ACG GAC AGA AGA -3'	
rrnB2	5'- C GAC GAC CAT GGC CAT TTG ATA TGC CTC CTA GCT GTG CAT CAG CGA CGT TTA ATA ATA TAA CAC CTG -3'	
yjbM3	5'- TCG GCT GTG GTC TAA AAC TAT TAA GCC TTT TTC TG -3'	
yjbM4	5'- GCA GCA GGA TCC CAT ACA TCC CCC AAT TCC GAA CCA GTT TCT ATA AAA AC -3'	
yjbM5	5'- GCA GCA AAG CTT TAG GTA AAG GGG AAG AAG AGC ATG AAA TTT GCC -3'	
yjbM6	5'- CGT ATT TGA CCG TCA CAT GCT CAC CGT TA -3'	
ywaC3	5'- CGT CCT CAT ACG TTA ACC GCA TCG TTT GTG -3'	
ywaC4	5'- GCA GCA GGA TCC GTT CGT CAT CTC CTT TAA CGG AAC TTT ATC CGC TG -3'	
ywaC5	5'- CGA CGA CTC GAG GAC GGC ACC CAA GTG CCG TCT TTT TTT ATT -3'	
ywaC6	5'- GGG CTA TCA AAA GGA CTT TAC CGC TGA CG -3'	
spo0H1	5'- TGG TTC CAT TAT GAG GAC AGC AGA TGC GG -3'	
spo0H2	5'- GCA GCA GGA TCC TCC GAT CCC CCC GGC GCA CGT AGA TAG -3'	
spo0H3	5'- GCA GCA AAG CTT GGA ATT TAT GCT ATA TTG ACA GTA TTT TTC TGA CTA TGA TAT GTT AC -3'	
spo0H4	5'- CGT TCA TCC ATG CCC ATT CTC TTC AGA ATG -3'	
codY3	5'- GGA GGC AGC CAG CCG AAT GGT GAA GAT -3'	
codY4	5'- GCA GCA GGA TCC CCT CCT AAA CAT TCC TCA TAT TAA ATT TTT CAC AAT ATA AAT TGA C -3'	
codY5	5'- GCA GCA GAA TTC GAG GGT TCT TTT TTT ATT TCA AAT AAA GGA AAT CAA TAA GCT TTA -3'	
codY6	5'- CCA GCA TTC CCG CTT CCG CTC ATT TGA GAA TT -3'	
A->G (1+2)	5'- C TTC TCA GAA TAC ATA CGG TAA AAT ATA CGG AAG AAG ATT TTT CGA CAA ATT CAC GTT T -3'	
GSP1-OA	5'- GCC TTA TGC TCT AAC CTC AGC -3'	
GSP2-0A	5'- GCA GCA GGA TCC ATC CGC AAC CAT TGC AAT GAA TTC ACT GTT GGT AGG -3'	
Seq+1-0A	5'- CGC AAG TCC GTC TAG ATG CGG CAT AA -3'	
luc2	5'- GCA GCA GGA TCC TTA CAC GGC GAT CTT TCC GCC CTT CTT GGC CTT TA -3'	
OAcorepromo	5'- CGA CGA GAA TTC TTC ACT TCT CAG AAT ACA TAC GGT AAA ATA TAC ATG CTT TTA TAT AGG GAA AAG GTG GTG AAC TAC TAT GGA AGA CGC CAA AAA CAT AAA GAA AGG CCC G -3'	

Restriction endonuclease sites are in bold letters.
Added nucleotide to conserve the phase between the promoter and the luciferase is underlined.
Mutageneised nucleotides are both in bold letters and underlined.
