# Supplementary material for: Fluctuations in spo0A Transcription Control Rare Developmental Transitions in Bacillus subtilis
Source: PLoS Genet. 2011 Apr 28;7(4):e1002048. doi: 10.1371/journal.pgen.1002048 (PMC3084206; doi:10.1371/journal.pgen.1002048)
Supplement: Text S1 — The method that allows us to measure the rate of transcription of a given gene during the growth of B. subtilis using the Firefly luciferase as a reporter gene is described in detail. (RTF) [file pgen.1002048.s009.rtf]

Fluctuations in spo0A transcription control rare developmental transitions in Bacillus subtilis:
Text S1

Nicolas Mirouze1, Peter Prepiak1 and David Dubnau1,2*
1Public Health Research Center 
2Department of Microbiology and Molecular Genetics
New Jersey Medical School
Newark, New Jersey
USA


Methods

Firefly luciferase as a reporter gene in Bacillus subtilis
We present data obtained using a powerful method that follows the rate of transcription of a given gene during the growth of B. subtilis. This technique, used previously in the model bacterium Streptococcus pneumoniae [1,2], employs the firefly (Photinus pyralis) luciferase as a reporter. This enzyme catalyses a reaction in which light is produced by the oxidation of luciferin in the presence of ATP. Luciferin is added to the culture medium and the cells provide ATP. Bacterial luciferase was previously used with B. subtilis [3].
We have found that the fusion of a promoter to the luciferase coding sequence, accompanied by measurement of light output and absorbance in a plate reader equipped for luminometry, permits real time measurement of both bacterial growth and the transcription rate from a promoter of interest. 
Figure S1A shows the light output obtained from a Pspo0A-luciferase fusion at two different times during the growth of a culture, as a function of the initial luciferin concentration. Saturation was achieved at about 1.5 mg/ml. In our experiments we therefore employed this concentration. Figure S1B shows that the rate of entry of luciferin into the cells does not limit our measurements, because, when the luciferin was added in the middle of growth, enough substrate had diffused into the cells within one minute, to give a result similar to that obtained when the luciferin was added at the beginning of the experiment. Probably because oxyluciferin (produced by the oxidation of the luciferin) inhibits the reaction, luciferin added later in the experiment produced a higher peak value of light output at a given point than when added earlier, suggesting that our values may be uniformly somewhat underestimated. However, this effect disappears within a few minutes and the curves corresponding to various times of addition are then essentially identical. In addition, we can conclude from this experiment that the stability of the luciferin is not affected in growing B. subtilis cultures.  Also, the consumption of ATP by the luciferase reaction has little impact on the global ATP pool of the cells because the addition of luciferin does not affect the growth curves (not shown). 
It is worth noting that the Km of firefly luciferase for ATP is about 100 μM [4], whereas growing Bacillus cells generally have ATP concentrations in the low mM range [5,6]. When B. subtilis was starved for glucose, the ATP pool decreased as expected for energy-starved cells (legend to Figure 6 in [7]). Nevertheless, in the diauxie experiment shown in our Figure 3, glucose starvation resulted in an increase in the transcription of spo0A suggesting that ATP did not limit light output in vivo. Finally, the correspondence between the decrease in light output from PabrB and the increase in spo0A transcription measured using the luciferase fusion (Figures 1A and B), strongly argues that the increase in Pspo0A activity during the growth pauses is real and is not an artifact of ATP concentration. 
Figures S1C and D show that when puromycin was added to cultures to inhibit the synthesis of luciferase from the spo0A promoter, light output rapidly decreased, suggesting that the luciferase protein is unstable in B. subtilis, with an apparent half life of about 6 minutes. We have repeated this experiment adding puromycin in stationary phase, with the same result (not shown). Our measurements therefore reflect transcription rate with a relatively small contribution from the cumulative effect of transcription. This particular characteristic of luciferase is in stark contrast with the behavior of other reporters, e.g. β-galactosidase, with which it is difficult to detect decreases in the rate of transcription, particularly if they are transient. 
Figure S2 shows that the method is extremely reproducible with respect to measurements of both growth and light output. 

Calculation of growth rates. 
Given two consecutive points in the growth curve (OD1, T1) and (OD2, T2), the slope or growth rate was defined as the ratio between the difference of the OD values (OD2-OD1) and the difference of the corresponding time (T2-T1). Changes in the growth rate are smoothed and presented in our graphs as a 'moving average' trend line determined using Excel, which indicates the changes in the slopes during growth.

Transcription of spo0A in various mutant backgrounds
Transcription from Pspo0A in additional mutant backgrounds is shown in Figures S4 and S5.


 References to supplement

1. Prudhomme M, Attaiech L, Sanchez G, Martin B, Claverys JP (2006) Antibiotic stress induces genetic transformability in the human pathogen Streptococcus pneumoniae. Science 313: 89-92.
2. Chastanet A, Prudhomme M, Claverys JP, Msadek T (2001) Regulation of Streptococcus pneumoniae clp genes and their role in competence development and stress survival. J Bacteriol 183: 7295-7307.
3. Carmi OA, Stewart GS, Ulitzur S, Kuhn J (1987) Use of bacterial luciferase to establish a promoter probe vehicle capable of nondestructive real-time analysis of gene expression in Bacillus spp. J Bacteriol 169: 2165-2170.
4. DeLuca M, McElroy WD (1984) Two kinetically distinguishable ATP sites in firefly luciferase. Biochem Biophys Res Commun 123: 764-770.
5. Guffanti AA, Clejan S, Falk LH, Hicks DB, Krulwich TA (1987) Isolation and characterization of uncoupler-resistant mutants of Bacillus subtilis. J Bacteriol 169: 4469-4478.
6. Jolliffe LK, Doyle RJ, Streips UN (1981) The energized membrane and cellular autolysis in Bacillus subtilis. Cell 25: 753-763.
7. Krasny L, Gourse RL (2004) An alternative strategy for bacterial ribosome synthesis: Bacillus subtilis rRNA transcription regulation. Embo J 23: 4473-4483.
8. Quisel JD, Burkholder WF, Grossman AD (2001) In vivo effects of sporulation kinases on mutant Spo0A proteins in Bacillus subtilis. J Bacteriol 183: 6573-6578.
9. Quisel JD, Grossman AD (2000) Control of sporulation gene expression in Bacillus subtilis by the chromosome partitioning proteins Soj (ParA) and Spo0J (ParB). J Bacteriol 182: 3446-3451.
10. Mandic-Mulec I, Gaur N, Bai U, Smith I (1992) Sin, a stage-specific repressor of cellular differentiation. J Bacteriol 174: 3561-3569.
11. Branda SS, Gonzalez-Pastor JE, Ben-Yehuda S, Losick R, Kolter R (2001) Fruiting body formation by Bacillus subtilis. Proc Natl Acad Sci U S A 98: 11621-11626.
12. Nanamiya H, Kasai K, Nozawa A, Yun CS, Narisawa T, et al. (2007) Identification and functional analysis of novel (p)ppGpp synthetase genes in Bacillus subtilis. Mol Microbiol 67: 291-304.
